# Host Cofactors and Pharmacologic Ligands Share an Essential Interface in HIV-1 Capsid That Is Lost upon Disassembly

**DOI:** 10.1371/journal.ppat.1004459

**Published:** 2014-10-30

**Authors:** Amanda J. Price, David A. Jacques, William A. McEwan, Adam J. Fletcher, Sebastian Essig, Jason W. Chin, Upul D. Halambage, Christopher Aiken, Leo C. James

**Affiliations:** 1 Medical Research Council Laboratory of Molecular Biology, Division of Protein and Nucleic Acid Chemistry, Cambridge, United Kingdom; 2 Department of Pathology, Microbiology and Immunology, Vanderbilt University School of Medicine, Nashville, Tennessee, United States of America; Duke University Medical Center, United States of America

## Abstract

The HIV-1 capsid is involved in all infectious steps from reverse transcription to integration site selection, and is the target of multiple host cell and pharmacologic ligands. However, structural studies have been limited to capsid monomers (CA), and the mechanistic basis for how these ligands influence infection is not well understood. Here we show that a multi-subunit interface formed exclusively within CA hexamers mediates binding to linear epitopes within cellular cofactors NUP153 and CPSF6, and is competed for by the antiretroviral compounds PF74 and BI-2. Each ligand is anchored via a shared phenylalanine-glycine (FG) motif to a pocket within the N-terminal domain of one monomer, and all but BI-2 also make essential interactions across the N-terminal domain: C-terminal domain (NTD:CTD) interface to a second monomer. Dissociation of hexamer into CA monomers prevents high affinity interaction with CPSF6 and PF74, and abolishes binding to NUP153. The second interface is conformationally dynamic, but binding of NUP153 or CPSF6 peptides is accommodated by only one conformation. NUP153 and CPSF6 have overlapping binding sites, but each makes unique CA interactions that, when mutated selectively, perturb cofactor dependency. These results reveal that multiple ligands share an overlapping interface in HIV-1 capsid that is lost upon viral disassembly.

## Author Summary

The early steps of HIV-1 infection are poorly understood, in part because of the difficulty in obtaining high-resolution information on encapsidated virus and its interaction with host cofactors. This, in turn, has made it difficult to design effective anti-capsid (CA) drugs. In our present study, we have used stabilized hexamers of HIV-1 CA to obtain complexed crystal structures with two cellular cofactors that are important for HIV-1 infection. These structures and accompanying virology reveal an essential interface in the capsid of HIV-1 that is lost upon viral uncoating. This interface is used to recruit both the nuclear targeting cofactor CPSF6 and NUP153, a nuclear pore component that facilitates nuclear entry. The high-resolution information provided by these structures reveals that the interface is degenerate and CA mutations can be made that selectively perturb sensitivity to each cofactor. This interface is also competed by two antiviral drugs, PF74 and BI-2, whose different mechanisms of action are not fully understood. We show that PF74, but not BI-2, binds across monomers within multimerized capsid affecting an inter-hexamer interface that is crucial for maintaining intact virions and that the addition of saturating concentrations of PF74 causes an irreversible block to viral reverse transcription.

## Introduction

The early events in HIV-1 infection proceed through a complex series of steps that include translocation of the viral core through the cytosol, reverse transcription, capsid uncoating, nuclear entry and integration. A number of host cofactors have been identified that regulate these processes, including cyclophilin A (CypA [1]), the karyopherin substrate CPSF6 [2], [3], nuclear import/pore proteins TNPO3, NUP153, NUP358 [4], [5], [6], and LEDGF [7]. Inhibition of CypA with cyclosporine reduces viral infectivity [8], promotes innate immune sensing [3] and alters the site of viral integration [9]. Depletion of TNPO3 [10], [11], NUP358 or NUP153 inhibits nuclear entry and infectivity and, at least in the case of TNPO3 and NUP358, alters integration site targeting [11], [12]. Finally, CPSF6 depletion inhibits infection of primary macrophages by revealing HIV-1 to innate immune sensing [3].

How the virus coordinates recruitment of these cofactors and how they influence each other is not well understood, but there is a growing body of data suggesting that the viral capsid plays a key role. Viral dependence on post-entry cofactors is dictated by CA whilst CA mutations affect all post-entry processes from reverse transcription to integration site selection [9], [10], [13], [14], [15]. CypA and NUP358 bind directly to an exposed loop in the CA NTD [16], [17] and loop mutations such as G89A or P90A that impair CA:CypA interactions phenocopy CypA deletion [18]. HIV-1 utilization of TNPO3 and NUP153 is also CA-dependent, and viruses with CA mutations such as N57A, Q63A/Q67A, K70R and N74D are insensitive to depletion of these proteins [19], [20]. These residues all define a recently identified protein-protein interface on CA that binds CPSF6, suggesting that CPSF6 may act upstream of other nuclear import cofactors and determine their usage [21]. However, CPSF6 binding to CA is critically dependent upon an ‘FG’ motif [21] and NUP153 has recently been shown to interact directly with viral cores using a similar motif [20].

While CPSF6 and NUP153 share a common ‘FG’ binding motif, their interactions with CA are distinct. For example, CA mutant N74D abolishes binding to CPSF6 [21] but not NUP153 [20]. Moreover, despite this different binding pattern, N74D escapes both CPSF6 and NUP153 dependence suggesting that CPSF6 and NUP153 have distinct roles. A role for NUP153 in HIV-1 nuclear entry is substantiated by depletion experiments in which the production of 2-LTR circles, which form only within the nucleus, and HIV-1 integration, are reduced [22]. CPSF6 may also have a role in nuclear import by recruiting TNPO3 to the virus and providing transport to active nuclear pores [23]. In support of this, it has been shown that TNPO3 is not required for infection when CPSF6 binding is abolished by the CA mutation N74D [2]. Further evidence for a link between CPSF6 and TNPO3 include data showing that mutants of TNPO3 that are selectively impaired for interaction with CPSF6 do not support HIV-1 infection [24], whilst TNPO3-depleted cells are refractory to wild-type HIV-1 infection only in the presence of endogenous CPSF6 [25]. Finally, the addition of a nuclear export sequence (NES) onto CPSF6 inhibited HIV-1 in TZM-bl cells [25]. However, the role of CPSF6 and Nup153 is complicated by the fact that perturbing their involvement gives rise to different phenotypes in different cell types. For instance, mutants T54A/N57A and Q63A/Q67A (which are sensitive to the synthetic restriction factor TRIM-CPSF6 but not TRIM-NUP153 [20]) have reduced infection in nondividing HeLa cells but only T54A/N57A has reduced infectivity in nondividing macrophages [26]. Meanwhile, neither depletion of CPSF6 [2] nor introduction of CPSF6 non-binding CA mutations N74D or T107A [19], [21] reduces infection of HIV-1 in single round replication assays in cell lines, whilst in macrophages N74D or CPSF6 depletion inhibits replication [3]. Importantly, it is not only changes in infection that are associated with an impaired ability of the capsid to engage CPSF6; N74D results in altered integration site targeting in HeLa cells [9].

The antiviral compounds PF-3450074 (PF74) [27] and BI-2 [28] have been shown to interact with the same pocket in the CA NTD as NUP153 and CPSF6. PF74 inhibits infection, blocks reverse transcription [29] and prevents replication in macrophages [3]. BI-2 has the same reported affinity for CA as PF74 yet does not block reverse transcription nor is as potent an inhibitor. These differences in reverse transcription inhibition are mirrored by NUP153 and CPSF6; NUP153 does not block reverse transcription but expression of a CPSF6 mutant lacking its nuclear localization sequence (hereafter referred to as ‘CPSF6ΔNLS’) inhibits viral DNA synthesis [3], [30]. While complexed crystal structures exist for the four ligands - NUP153, CPSF6, PF74 and BI-1 (a BI-2 precursor) - these structures provide no mechanistic basis to explain their differing effects on viral infection [21],[27],[28]. One potential explanation for this is that all three structures were solved using monomeric CA NTD, whereas a fully assembled HIV-1 capsid is comprised exclusively of multimeric CA, predominately hexamers, containing both NTD-NTD and NTD-CTD interfaces [31]. Understanding the effect of ligand binding on intact capsid is of particular interest as there are conflicting reports on the effect of PF74 and BI-2 on capsid stability [28], [29], [32].

Here we report crystal structures of assembled HIV-1 CA hexamers with peptides comprising the linear binding epitopes of NUP153 and CPSF6 and the two antiviral compounds PF74 and BI-2. These structures reveal that the first three ligands make interactions both within and between CA subunits, using a previously unknown binding site at the NTD-CTD interface that significantly increases binding to CPSF6 and PF74 and is required for NUP153 interaction. NUP153, CPSF6 and PF74 utilize a distinct sub-set of residues within this site, providing a molecular basis for their associations with capsid and the resulting effects on HIV-1 infection.

## Results

### CPSF6, NUP153 and PF74 exhibit preferential binding to hexameric CA

**Figure 1:**
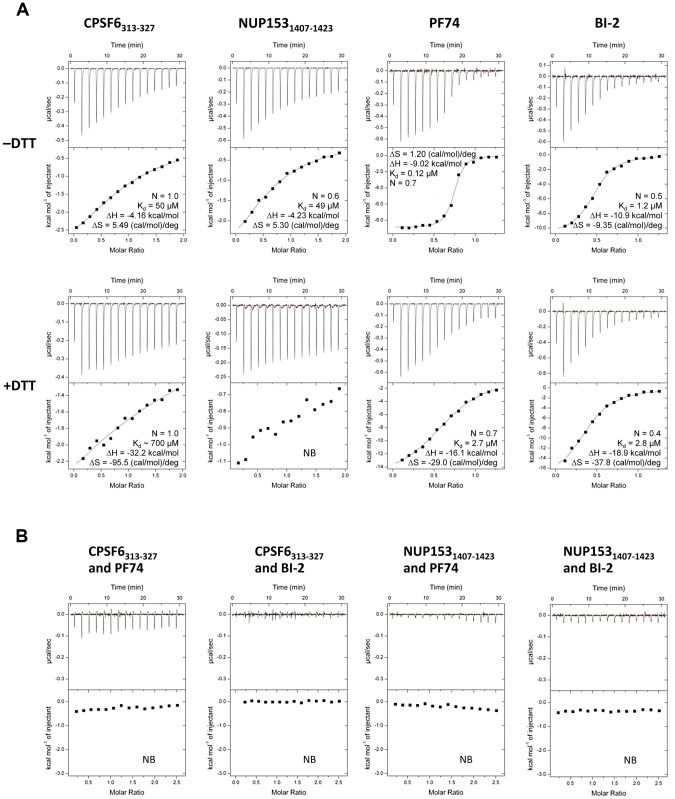
Binding of HIV-1 hexamer to CPSF6, NUP153, PF74 and BI-2. (A) ITC binding isotherms of HIV-1 hexamer titrated into different ligands in either the presence or absence of DTT. For both CPSF6 and NUP153, a peptide corresponding to residues 313–327 or 1407–1423 respectively was used. (B) Titration of HIV-1 cofactors CPSF6 and NUP153 into hexamer in the presence of pharmacologic inhibitors. Thermodynamic parameters are indicated for interactions displaying a clear binding isotherm; approximate affinities are shown for weak interactions; NB = no binding detectable.

CPSF6, PF74 and BI-2 have all been shown to interact directly with HIV-1 CA NTD by isothermal titration calorimetry (ITC) [21], [27], [28], whilst NUP153 from cell lysate has been shown to co-sediment with His-tagged CA protein [20]. Crystal structures of CPSF6, PF74 and BI-2 bound to monomeric CA-NTD all show interaction with the same binding site, at the base of the CypA-binding loop and between helices 3, 4 and 5. However, the phenotypes observed upon binding to each ligand are different. NUP153 depletion does not affect reverse transcription but blocks nuclear entry [22]. Expression of CPSF6 lacking its nuclear localisation sequence (CPSF6ΔNLS) blocks reverse transcription [3], [30], as does PF74, while BI-2 inhibits infection post-reverse transcription [28], [29], [30]. To attempt to explain how these diverse phenotypes are achieved, we carried out interaction studies using a CA that can be assembled into stable hexamers through the formation of engineered disulfide bonds at the NTD-NTD intrahexameric interface [33]. In each case we compared binding to monomers by reducing hexamers with DTT. Previously, we have shown that a 15-mer peptide from CPSF6 containing a central FG motif is sufficient for binding to CA NTD [21]. Using a similar strategy, an FG-containing 17-mer from NUP153 was synthesized, based on published co-sedimentation data [20]. The two peptides (CPSF6_313–327_ with sequence PVLFPGQPFGQPPLG and NUP153_1407–1423_ with sequence TNNSPSGVFTFGANSST) were then tested for binding to intact and dissociated CA hexamer alongside PF74 and BI-2. Unless otherwise stated, all experiments describing ‘CPSF6’ or ‘NUP153’ refer to these peptides. Binding of all four ligands was observed to monomeric CA, confirming previous monomer studies with CPSF6_313–327_, PF74 and BI-2 and showing that the NUP153_1407–1423_ peptide is sufficient for direct binding (Figure 1A). Comparison to matched data with hexamer however reveals that, with the exception of BI-2, each ligand interacts with significantly improved affinity to hexameric CA compared with monomeric CA. CPSF6 binds with a 14-fold increased affinity to hexamer and PF74 with a 22-fold increase. This difference was most dramatic with NUP153, which showed comparable affinity to the hexamer as CPSF6 but negligible binding to monomeric CA. This result is in contrast to predictions that oligomerization does not affect NUP153 binding and that there is efficient binding to monomeric CA [20]. This is a particularly significant result because it suggests that HIV-1 docks to the nuclear pore as some form of assembled capsid. Although the multimerisation status of capsid at the nuclear pore is unknown, our data indicates that if the capsid disassembles prior to locating to the nuclear pore then the ability of HIV-1 to engage NUP153 would be significantly compromised.

Comparison of the affinities with which the different ligands bind CA reveals several important points. First, CPSF6 and NUP153 bind hexamer with identical affinities, although binding to monomer is different suggesting that they make different interactions. Second, PF74 and BI-2 have identical affinities to monomeric capsid but PF74 binds hexamer with a 10-fold higher affinity than BI-2. Indeed, BI-2 binds hexamer with similar affinity to monomer (within 2-fold; Figure 1A). Third, the finding that PF74 actually binds capsid with an affinity of ∼0.1 µM explains why its potency in infection experiments is significantly greater than its published affinity. Previously, we have shown that PF74 competes with CPSF6 for binding to monomeric NTD CA [21]. Competition experiments show that both PF74 and BI-2 also inhibit CPSF6 binding to hexamer (Figure 1B). Furthermore, PF74 and BI-2 also prevent NUP153 binding to hexamer, suggesting that the drugs are competitive inhibitors of both host cofactors (Figure 1B).

### Cofactors CPSF6 and NUP153 bind a hexamer-specific interface formed between two adjacent monomers

**Figure 2:**
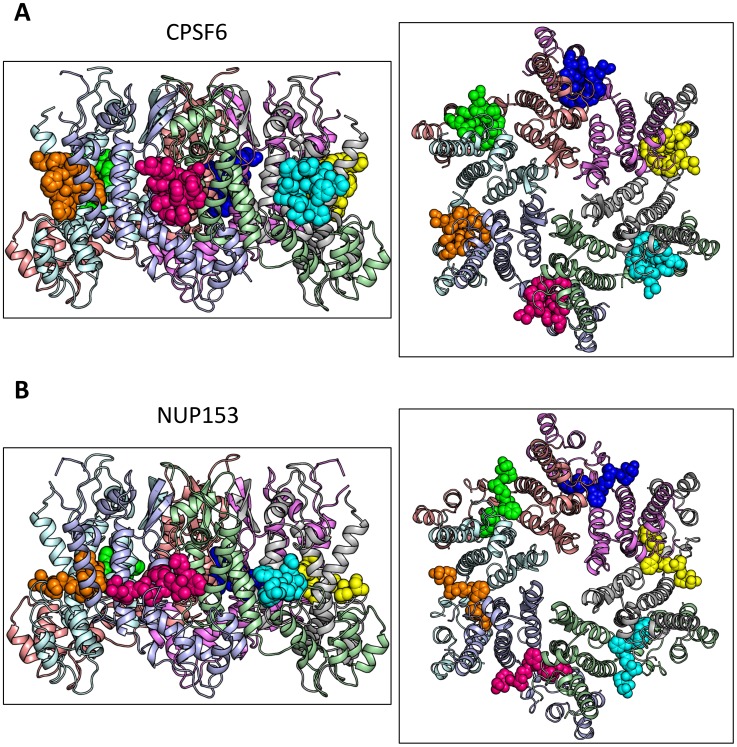
Crystal structures of CPSF6_313–327_ (A) and NUP153_1407–1423_ (B) FG-containing peptides in complex with HIV-1 hexamer. The P6 crystal form is shown, with the six subunits of the HIV-1 capsid generated by crystallographic symmetry. The capsid is shown in a ribbon representation with each monomer colored differently. Atoms comprising the cofactor peptides are shown as spheres, with each peptide colored separately. There are six cofactor binding sites per hexamer and six bound cofactor peptides. For each structure two views are shown, related by 90**°**.

**Figure 3:**
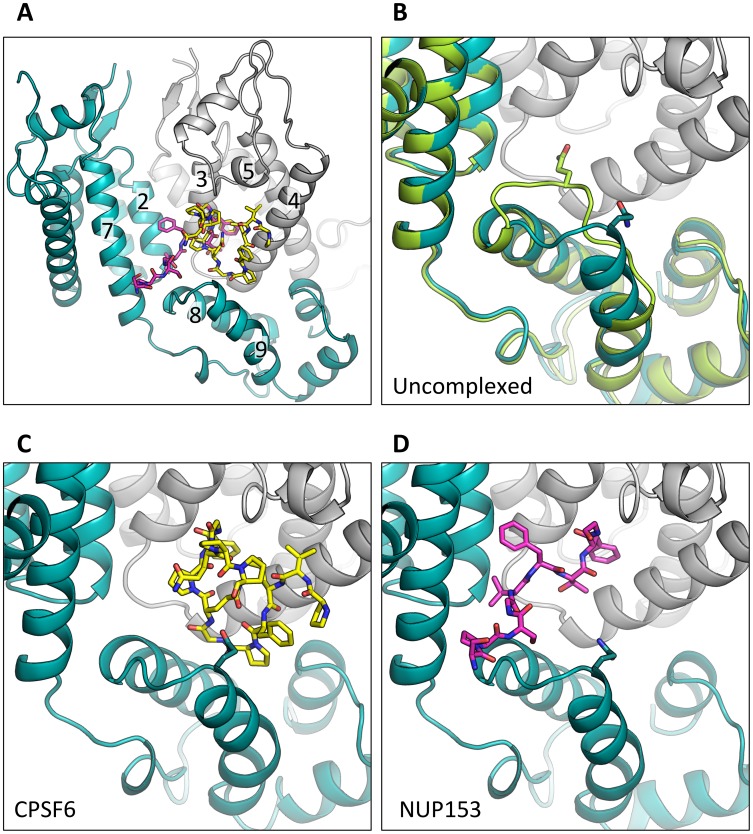
CPSF6 and NUP153 bind a multi-subunit interface in HIV-1 hexamer. (A) Two monomers from the CPSF6 P2_1_2_1_2_1_ complexed structure are shown in gray and teal, with the CPSF6 peptide in yellow and the NUP153 peptide from the P2_1_2_1_2_1_ complexed structure superposed and shown in pink. The helices that comprise the binding site are numbered. (B) Two monomers from the uncomplexed P2_1_2_1_2_1_ hexamer structure (3H4E) showing representative helices 8 and 9 in ‘open’ (teal) and ‘closed’ (green) states are superposed. The side-chain of residue Q179 is shown in stick representation. (C) CPSF6 binding site in the P2_1_2_1_2_1_ structure, with the peptide shown in yellow and subunits colored as above. (D) NUP153 binding site in the P2_1_2_1_2_1_ structure, with peptide shown in pink and subunits colored as above. CPSF6 and NUP153 preferentially bind an open conformation of helices 8 and 9.

To understand how CPSF6 and NUP153 interact with hexameric CA we determined X-ray crystal structures with the same peptide ligands used in the ITC studies. Crystals of each complex were obtained in two different spacegroups, hexagonal (P6) and orthorhombic (P2_1_2_1_2_1_) (Table 1), similar to those reported for the uncomplexed CA hexamer [31]. The structures reveal that the binding pocket identified for CPSF6 in CA NTD is actually part of a much larger protein-protein interface that also accommodates NUP153 and is present only in assembled CA (Figure 2 and 3). The complete binding site is formed by NTD helices 3 and 4 of one CA monomer (referred to as the ‘first site’) and NTD helices 2 and 7 and CTD helices 8 and 9 from an adjacent monomer (referred to as the ‘second site’). The cofactor interface incorporates a structurally dynamic region involving CTD helices 8 and 9 that was observed to adopt two distinct conformations in the orthorhombic crystal structure of the uncomplexed hexamer (Figure 3). Comparison to CPSF6 and NUP153 complexes solved in the same orthorhombic form reveals that these cofactors selectively bind to one of these conformations, which we have termed ‘open’ (Figure 3 and Supplementary Figure S1). In the ‘open’ conformation, helices 8 and 9 are of equal length and separated by a single residue. In the alternative ‘closed’ conformation ∼1/3 of helix 9 has unwound, creating a loop of six residues (177–182) that folds back into the binding site, partially occluding it. Binding of CPSF6 and NUP153 to hexamer in the ‘closed’ conformation is prevented by steric clashes with loop_177–182_. A consequence of cofactor binding may therefore be to alter capsid conformational dynamics and drive equilibrium towards an ‘open’ conformation at the CTD-CTD or hexamer-hexamer interface.

**Table 1:** Data collection and refinement statistics.

	Hex-CPSF6 (Hexagonal)	Hex-CPSF6 (Orthorhombic)	Hex-Nup153 (Hexagonal)	Hex-Nup153 (Orthorhombic)	Hex-PF74 (Hexagonal)	Hex-BI2 (Hexagonal)
**Data collection**						
Space group	P 6	P 2_1_2_1_2_1_	P 6	P 2_1_2_1_2_1_	P 6	P 6
Cell dimensions						
* a*, *b*, *c* (Å)	91.5, 91.5, 57.0	135.1, 135.9, 208.2	91.5, 91.5, 57.0	135.4, 138.1, 211.9	91.2, 91.2, 56.3	90.9, 90.9, 56.6
α, β, γ (°)	90, 90, 120	90, 90, 90	90, 90, 120	90, 90, 90	90, 90, 120	90, 90, 120
Resolution (Å)	57.00-2.05 (2.16-2.05)	48.60-2.80 (2.95-2.80)	35.67-1.77 (1.81-1.77)	46.07-3.00 (3.06-3.00)	45.87-2.03 (2.14-2.03)	14.87-2.21 (2.28-2.21)
*R*_merge_	0.266 (0.973)	0.069 (0.588)	0.101 (0.909)	0.085 (0.581)	0.186 (0.843)	0.164 (0.451)
*I*/σ*I*	4.6 (2.0)	11.7 (2.2)	7.3 (1.6)	8.6 (1.7)	7.7 (2.0)	7.2 (3.0)
Completeness (%)	100.0 (100.0)	95.1 (97.6)	95.1 (95.8)	97.0 (97.3)	98.1 (87.0)	93.4 (72.5)
Redundancy	7.3 (6.8)	3.6 (3.6)	3.2 (3.2)	3.2 (3.1)	6.0 (5.5)	3.9 (3.4)
**Refinement**						
Resolution (Å)	57.00-2.05 (2.10-2.05)	48.60-2.80 (2.87-2.80)	35.67-1.77 (1.82-1.77)	46.07-3.00 (3.08-3.00)	45.87-2.04 (2.09-2.04)	14.87-2.22 (2.28-2.22)
No. reflections	16208 (1135)	84854 (6359)	23742 (1742)	73490 (5439)	16167 (1156)	11864 (817)
*R*_work_/*R*_free_	0.212/0.252 (0.314/0.329)	0.230/0.262 (0.331/0.381)	0.194/0.211 (0.290/0.313)	0.247/0.267 (0.364/0.387)	0.198/0.239 (0.317/0.343)	0.242/0.270 (0.400/0.481)
No. atoms						
Protein	1701	19958	1710	19269	1608	1565
Ligand/ion	1	-	-	1	33	26
Water	147	-	200	-	148	76
*B*-factors						
Protein	21.5	87.0	24.3	78.8	30.0	36.6
Ligand/ion	20.5	-	-	58.1	21.8	42.5
Water	32.3	-	33.7	-	33.4	33.5
R.m.s. deviations						
Bond lengths (Å)	0.004	0.004	0.005	0.004	0.005	0.006
Bond angles (°)	0.837	0.739	0.877	0.662	0.924	0.918

Statistics are given for the six structures, titled by complex followed by the spacegroup in parentheses. Values in parentheses are for the highest-resolution shell.

The ‘open’ and ‘closed’ conformations of the co-factor binding site represent two alternate states of the hexamer. The ‘open’ conformation comprises a stable helix-loop-helix motif whereas the ‘closed’ conformation is held in place by R173 through a planar stacking interaction with Q179 and hydrogen bond with N57. A conformationally dynamic interface will influence the entropy of binding, therefore the complexation of hexamer with CPSF6, NUP153 and PF74 would be expected to occur with a different change in entropy than BI-2. Examination of the ITC data reveals that this is indeed the case and binding of BI-2 occurs with a negative ΔS whereas it is positive with the other ligands (Figure 1A). This interpretation is supported by NMR experiments, which show that loop_177–182_ isomerizes between two discrete conformers [34]. The two conformations are not equally energetically stable and one of the conformers is only transiently populated (∼7% at equilibrium).

**Figure 4:**
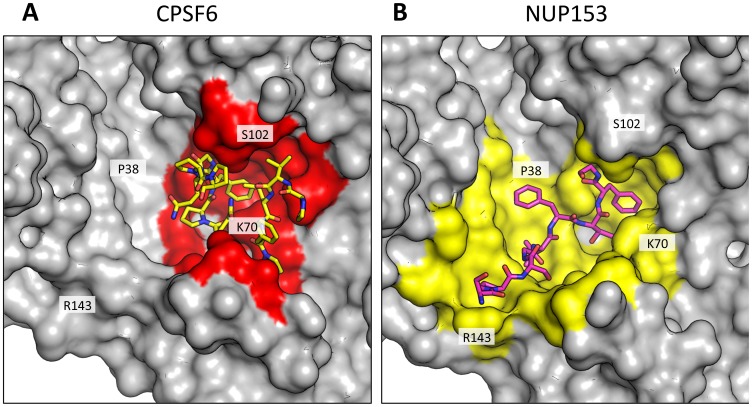
CPSF6 and NUP153 interact with distinct sets of CA residues within the hexamer interface. A molecular surface representation of the CPSF6 and NUP153 binding site in hexameric capsid is shown in gray. The interface is formed between two neighbouring monomers and is shown in a similar orientation to Figure 3. The CPSF6 (A) and NUP153 (B) peptides in the different complexed structures are shown in yellow and pink respectively. The binding ‘footprint’ of each ligand is shown in red for CPSF6 and yellow for NUP153. The footprint was calculated using PISA [44] and is defined as residues containing atoms that become desolvated upon binding (i.e. are within 1.4 Å of the ligand).

**Figure 5:**
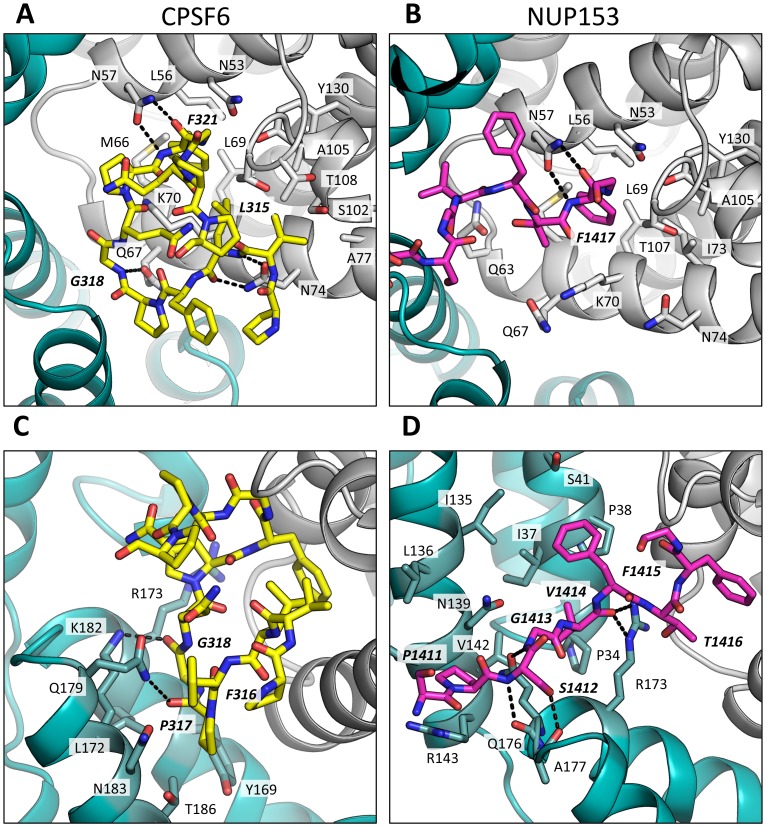
CPSF6 and NUP153 interact both within and between CA monomers. Detailed views of the interactions between the ligands and the binding site within hexameric capsid are shown. In each case two adjacent monomers of the hexamer are colored gray and teal, with the side chains of specific contacting residues displayed and labelled in standard text. Potential hydrogen bonds with ligands are indicated by dashed lines. The upper panels (A and B) focus on interactions that occur in the first binding site (within one monomer), while the lower panels (C and D), show interactions in the second binding site (with the second monomer). CPSF6 is shown in yellow and NUP153 in pink, with important ligand residues labelled in bold and italic text.

CPSF6 and NUP153 have been identified as disparate host cofactors that nevertheless share a common binding site on HIV-1 CA. However, whilst they share interactions within the first, monomeric, binding site they make distinct interactions to the second site, in the context of hexameric CA (Figure 4). Within one monomer, CPSF6 interacts with N53, L56, N57, M66, Q67, L69, K70, I73, N74, A77, S102, A105, G106, T107, T108 and Y130 (Figure 5A). Of these interactions, binding and restriction studies have confirmed an important role for N57, M66, K70, N74 and T107 [21]. Meanwhile, NUP153 interacts with residues N53, L56, N57, Q63, M66, Q67, L69, K70, I73, A105, G106, T107 and Y130 (Figure 5B). There was no observed interaction between NUP153 and CA N74, supporting findings that mutation N74D specifically abolishes CPSF6 binding [20], [21]. The overlap in residue usage reflects similarities in the way in which CPSF6 and NUP153 engage capsid within the monomeric binding site. Previously, we have shown that CPSF6 residue F321, which occupies the same pocket as the phenyl rings of PF74 and BI-2, is critical for interaction of CPSF6 with capsid [21]. We confirmed that CPSF6 F321 is critical for hexamer binding, as mutation to alanine abolishes interaction (Supplementary Figure S2). In NUP153, an equivalent interaction is mediated by F1417, which superposes closely with CPSF6 F321 (Figure 3A and Figure 5A and B). ITC binding experiments confirm that this NUP153 residue is essential, as F1417A has no measurable binding to hexamer (Supplementary Figure S2). The importance of F321 in CPSF6 and F1417 in NUP153 is in part to orient the main-chain for hydrogen bond interactions with the side-chain of N57 (Figure 5A and B). This most likely explains why capsid mutant N57A was identified as critical for co-immunoprecipitation of NUP153 with CA NTD and for restriction by a TRIM-NUP153 fusion [20]. Within the monomer binding site, NUP153 makes unique contacts with Q63, while CPSF6 makes unique contacts with N74, A77, S102 and T108 (Figure 5A and B). The unique interaction of CPSF6 with N74 explains why mutation N74D prevents binding to CPSF6 but allows co-immunoprecipitation with NUP153 and remains sensitive to TRIM-NUP153 restriction [20].

**Figure 6:**
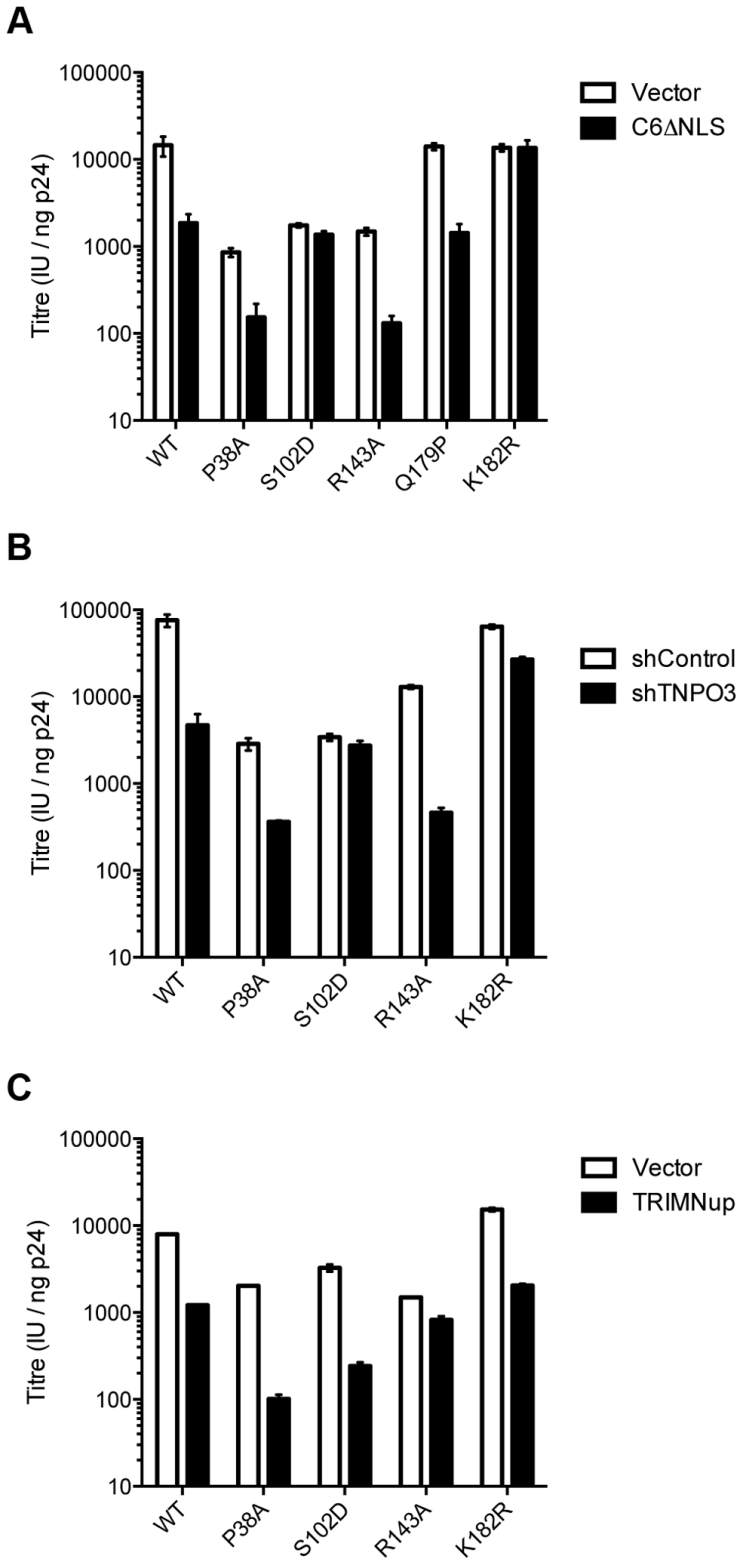
Sensitivity of CA mutants to restriction by CPSF6ΔNLS or TRIM-NUP153 or depletion of TNPO3. Titres of VSV-G pseudotyped GFP-encoding HIV-1 vectors bearing wild type or mutant CA on HeLa cells expressing empty vector (EV) or CPSF6ΔNLS (A), control knockdown cells (shControl) or cells depleted of TNPO3 (shTNPO3) (B), control knockdown cells (shControl) or cells expressing empty vector (EV) or TRIM-NUP153 (C). In each case infectivity is plotted as infectious units (IU)/ng p24. The data are representative of two independent experiments, each using three different virus doses.

Outside of the phenylalanine binding pocket, the two ligands form distinct interactions with hexameric capsid. Both cofactors make interactions across neighbouring hexamers but while CPSF6 contacts the CTD of the second monomer, NUP153 interacts predominantly with the NTD (Figures 3 and 5). The ligands diverge after the shared phenylalanine, with CPSF6 residues 315–319 intercalating between helices 4 and 9 in neighbouring monomers and making contacts with residues Y169, L172, R173, Q179, K182, N183 and T186, including hydrogen bonds with Q179 and K182 (Figure 5C). As these second site contacts are driven by CPSF6 residues that interact via their main-chain or through hydrophobic burial, their importance is difficult to assess through side-chain mutation. However, mutants F316A and P317A both have significantly reduced binding affinity for hexamer as measured by ITC, while the introduction of charged residues by either P317D or G318R abolishes binding completely (Supplementary Figure S2). The dramatic effect of G318 mutation on CPSF6 binding correlates with the reduced ability of NLS deleted CPSF6 mutant G318A to restrict HIV-1 [35]. To complement our studies using different CPSF6 peptides, we tested the sensitivity of interface mutants to restriction by CPSF6ΔNLS. Previously, we have shown that CPSF6 interface mutants N57A, Q67A, K70A, N74D and T107A are all resistant to restriction by CPSF6ΔNLS [21]. Of the remaining interface residues, A77D and T108A were noninfectious whilst S102D and K182R escaped restriction (Figure 6A). Q179P was still sensitive to restriction by CPSF6ΔNLS; however, its hydrogen bond to the carbonyl oxygen of P317 may simply have been replaced by a hydrogen bond with the nearby side-chain of N183, thus maintaining interaction.

Unlike CPSF6_313–327_, which forms almost a cyclised structure, NUP153 residues 1410–1417 form an extended linear conformation that runs from F1417 across the face of CA helices 2 and 7 from the second monomer. NUP153 interacts with helix 2 residues P34, I37, P38 and S41 and helix 7 residues I135, L136, N139, K140, V142 and R143. NUP153 also interacts with the end of helix 8, where it meets helices 2 and 7, and hydrogen bonds with R173, Q176 and A177. Interactions between NUP153 and the second monomer are essential, as shown by the ITC experiments in which NUP153 binds hexamer but not monomer (Figure 1A). NUP153 residues T1416, F1415, S1412 and P1411 all make important interactions with the second site. The side-chain of T1416 interacts directly with CA R173 and with CA Q63 via a water and T1416A abolishes binding by ITC (Supplementary Figure S2 and 3). F1415 forms a hydrophobic interaction with CA P38 and loss of this interaction (F1415A) also prevents binding. S1412 forms potential hydrogen bonds with CA A177 and Q176. Mutation S1412A has little impact on the affinity to hexamer but this is not unexpected as Q176 interaction is via the main-chain (Supplementary Figure S2). Finally, P1411 forms a hydrophobic interaction with the side-chain of R143 and mutation P1411A decreases binding by approximately 10-fold (Supplementary Figure S2). Only the central 9 residues of the NUP153 peptide have a rigid structure in the complexed crystal structure (1407-TNN**SPSGVFTFG**ANSST-1423). This suggests that the chosen peptide is sufficient to define the important contacting residues within this linear epitope. We cannot rule out that there may be other contacts outside of this region in the context of full-length NUP153 protein, however our data together with published work [20] suggests that a short FG-containing sequence is both necessary and sufficient for binding. In this respect, NUP153 and CPSF6 are similar in using a linear binding motif to interact with hexameric CA, anchored around a core phenylalanine but otherwise mediating diverse contacts.

The fact that CPSF6 and NUP153 make specific contacts with hexameric CA suggests that it is possible to selectively abolish binding to each ligand, in a similar manner to mutation N74D in the first site. Within the second site, NUP153 makes specific contacts with capsid residues P38 and R143, while CPSF6 makes specific contacts with S102 and K182. We mutated these residues and tested their sensitivity to restriction by CPSF6ΔNLS and TRIM-NUP153 and their dependence on TNPO3. NUP153 specific mutants P38A and R143A retained sensitivity to both CPSF6ΔNLS restriction and TNPO3 dependence (Figure 6A&B). However, CPSF6-specific mutants S102D and K182R rescued virus from both CPSF6ΔNLS restriction and TNPO3 dependence (Figure 6A&B). Meanwhile, mutants S102D and K182R were sensitive to TRIM-NUP153 while R143A escaped restriction (Figure 6C). In agreement with published data, mutant P38A had increased sensitivity to TRIM-NUP153, suggesting that the alanine substitution enhances binding (Figure 6C and [20]). These results confirm that P38A, S102D, R143A and K182R are cofactor selective mutants. Furthermore, mutants R143A and K182R support the functional importance of the second site for NUP153 and CPSF6 interaction respectively.

### PF74 and BI-2 have distinct binding mechanisms

**Figure 7:**
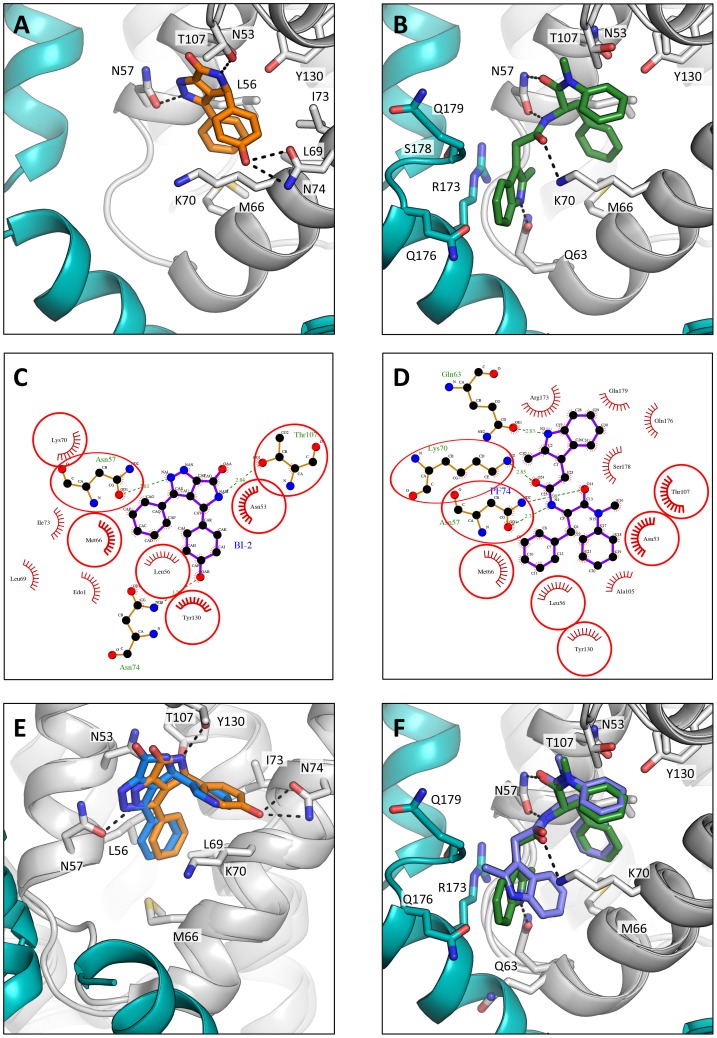
HIV-1 CA hexamer complexes with pharmacologic ligands BI-2 and PF74. Two monomers from hexameric structures complexed with BI-2 (A) and PF74 (B) are shown in a close-up view of the binding site, with one monomer in gray and a second monomer in teal. BI-2 is shown in orange and PF74 in green. Important CA interacting residues are shown as sticks and putative hydrogen bonds indicated by dashed lines. Ligplots [45] are shown for BI-2 (C) and PF74 (D) in which a ball-and-stick representation of the ligand is shown together with CA residues that contribute a hydrogen bond. Other interacting CA residues are indicated with a red-dashed semi-circle. Circled areas indicate interacting CA residues that are shared between both structures. (E) Superposition of the BI-2:hexamer complex (BI-2 in orange) with the previously solved BI-1:CA-NTD complex [28] (BI-1 in blue). The secondary structure of the superposed monomers from each structure is shown in gray, while capsid side-chains from only the hexamer complex are depicted. The adjacent monomer in the hexamer complex is shown in teal. (F) Superposition of the PF74:hexamer complex (PF74 in green) with the previously solved PF74:CA-NTD complex [27] (PF74 in slate). The capsids are colored as in (E).

The discovery that the binding site for CPSF6 and NUP153 is actually an extended interface that is formed at the junction between two monomers in the hexamer suggested that the interaction with PF74 and BI-2 should be re-examined. In particular, we wondered whether the two drugs engage hexamer differently, which would explain the higher affinity of PF74 and its different activity. Complexed structures of each drug with hexamer were solved in a P6 spacegroup and their mode of binding compared (Table 1). As expected, each has a phenyl ring that superposes almost exactly within the previously identified pocket in the NTD, making an equivalent interaction to F321 in CPSF6 and F1417 in NUP153 (Figures 5 and 7). However, this motif and hydrogen bonds with the side-chain of N57 are the only features shared by all four ligands. Uniquely amongst the ligands, BI-2 interacts solely within the NTD of each subunit (Figure 7A & C). Accordingly, the hexamer:BI-2 complexed structure is very similar to that of the published monomer structure with the related compound BI-1 [28]. The most significant difference between the two is that the hydroxyphenyl moiety in BI-2, which replaces the piperidine ring in BI-1, facilitates hydrogen bonding to N74 (Figure 7E). This BI-2 specific interaction may explain why it binds almost 10-fold more tightly than BI-1 to CA NTD [28]. Most significantly, the absence of any BI-2 interactions outside of the monomer explains why it does not bind hexamer with a substantially higher affinity, unlike PF74.

Analysis of the hexamer:PF74 structure reveals that, in addition to making contacts within the NTD of one monomer, PF74 interacts with the CTD of an adjacent monomer (Figure 7B & D). This additional interaction was unexpected and could not have been predicted by modelling the structure of PF74:CA NTD complex structure (pdb 2XDE) onto the hexamer structure, because the orientation of the 2-methylindole moiety has changed in the hexamer complex (Figure 7F). As a result of this reorientation, the 2-methylindole moiety forms a planar stacking interaction with the side-chain of R173 (Figure 7B). Cation-π interactions such as those between arginine and tryptophan can contribute significantly to binding energy, and computational studies suggest that in a solvent-accessible environment they are twice as strong as a typical salt bridge [36]. This indole re-orientation not only allows stacking with R173, it also reveals a hydrogen bond between the indole NH and Q63 (Figure 7B). Whether interaction between Q63 and PF74, despite being absent in the monomeric crystal structure, occurs during binding of PF74 to monomeric CA is unclear; there may be a requirement for hexamer both to supply R173 to orient the 2-methylindole and to position the side-chain of Q63 for hydrogen bonding to PF74. Together, these extra interactions seen in the context of the hexameric complex structure most likely account for the significant increase in affinity seen on binding of PF74 to hexamer *vs.* monomeric CA (Figure 1A).

As with the P6 structures of uncomplexed hexamer [31] and hexamer in complex with the peptide ligands (Supplementary Figure S1), the CTD region around the N-terminus of helix 9 was poorly ordered in the P6 hexamer:drug structures (Figure 7). Nevertheless, superposition of the P6 hexamer:PF74 structure on the orthorhombic uncomplexed hexamer structure showed that the interaction between PF74 and CA directly displaces the stacking interaction between R173 and Q179 that stabilises the ‘closed’ interface conformation, with the 2-methylindole taking the place of the glutamine side-chain. For this reason, PF74, like CPSF6 and NUP153, cannot bind to the hexamer when CTD helices 8 and 9 are in the ‘closed’ conformation (Supplementary Figure S4), although we cannot rule out the possibility of additional interactions between this region and the drug. In contrast, the closed conformation is not predicted to occlude BI-2 binding.

**Figure 8:**
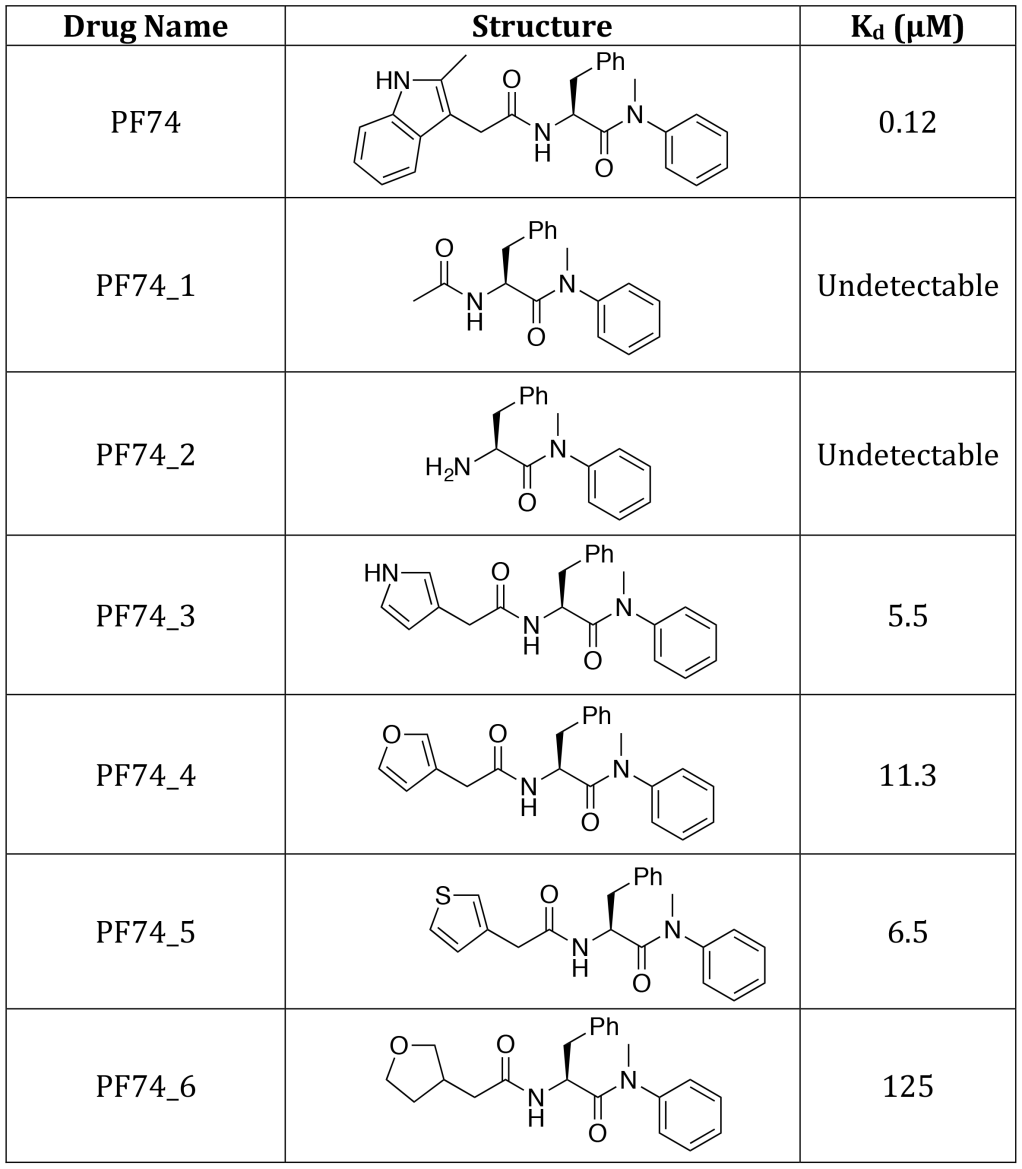
Structures of PF74 derivatives used in this study and their affinities of binding to CA. Affinities of binding to hexamer as measured by ITC are shown. No binding was detected between PF74_1 and PF74_2 to HIV-1 CA NTD, therefore the interaction with hexamer was not tested.

The different PF74 orientations in the monomer versus hexamer structures as well as the resulting new interactions raise the question of what capsid contacts are essential for drug binding. To investigate this we adopted a chemical genetics approach to avoid the pleiotropic effects of capsid mutants. We first attempted to assess the contribution of the 2-methylindole group by removing it either before or after the amine. No binding to CA NTD could be observed by ITC for either compound lacking the 2-methylindole (Figure 8, PF74_1 and PF74_2). This result suggests that hydrogen bonding to N57 is not sufficient for CA interaction and that Q63 and R173 are important for PF74 binding. To dissect the contributions of interaction with Q63 and R173 we replaced the 2-methylindole with pyrrole, furan, thiophene and tetrahydrofuran moieties that have altered aromaticity and hydrogen bond propensities (Figure 8). The pyrrole-containing compound (PF74_3) lacks the 6-membered ring found in 2-methylindole but retains the hydrogen-bond donor. This compound had a 46-fold reduction in affinity to hexamer, indicating that much of the enhanced binding to hexamer was lost (Figure 8; PF74_3). The furan and thiophene (PF74_4 and 5) compounds, which have a hydrogen bond acceptor instead of donor, interacted with hexamer with similarly weakened affinities as the pyrrole (a reduction of 94-fold and 54-fold respectively). This is probably because the electronegative element coordinates a glutamine (Q63), which is able to hydrogen-bond to both hydrogen donors (N) and hydrogen acceptors (O, S). Finally, the tetrahydrofuran derivative has lost aromaticity and therefore the ability to form cation-π interactions and has the weakest affinity for hexamer, reduced by >1000-fold. These results suggest that the binding of PF74 to hexamer is mediated primarily by a stacking interaction with R173.

**Figure 9:**
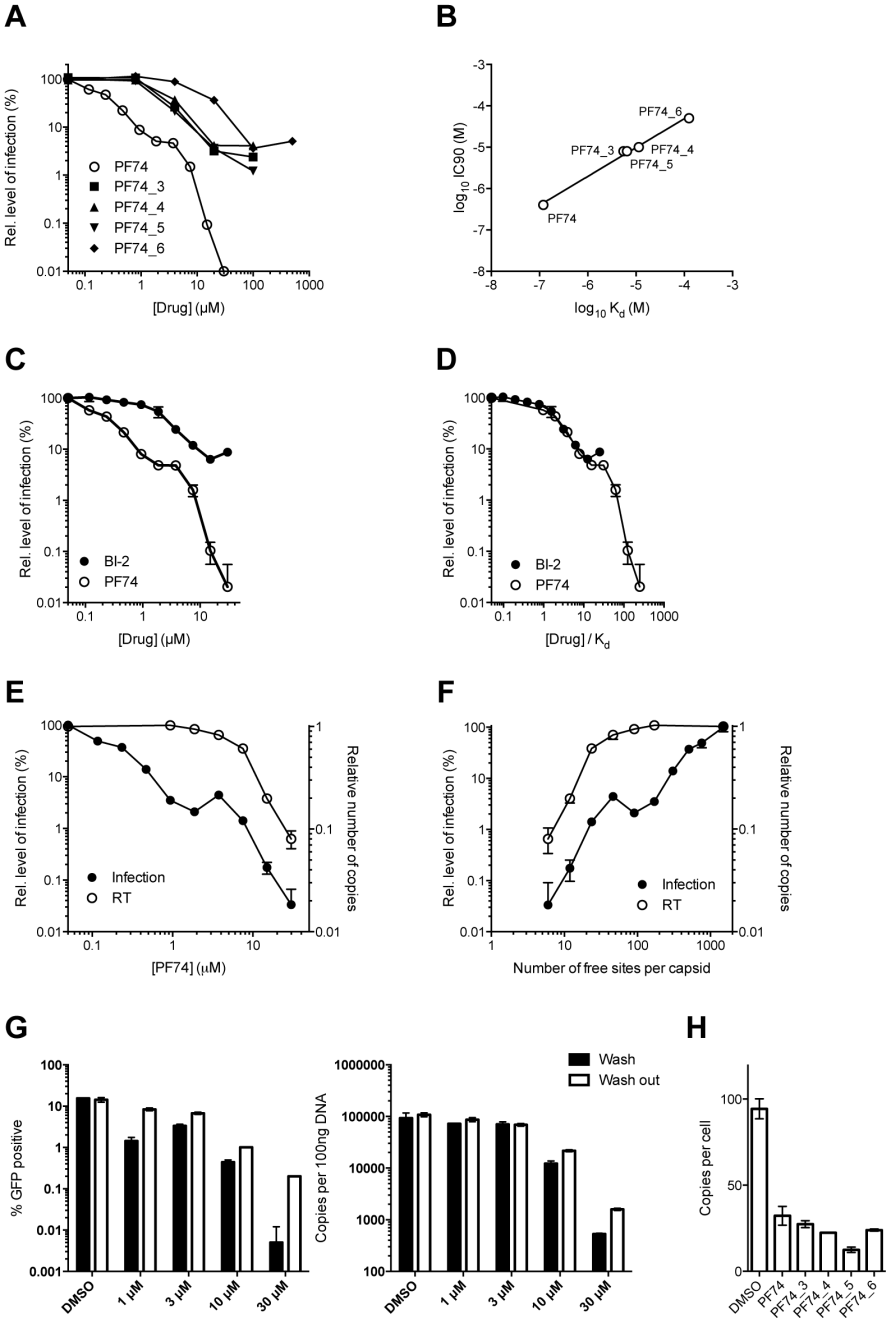
Inhibition of HIV-1 infection by BI-2, PF74 and PF74 derivatives. (A) Titration of PF74 or indicated derivatives onto HeLa cells infected with VSV-G pseudotyped GFP-encoding HIV-1 vector. For each titration, infectivity is normalised to the level in the absence of inhibitor (100%). (B) Correlation between the IC90 values derived from (A) and the Kd values for purified hexameric capsid as calculated by ITC (Figure 8). The values are highly correlative with a Pearson correlation coefficient of 0.9928 and a P-value of 0.0007. (C) Titration of PF74 or BI-2 onto cells infected with VSV-G pseudotyped GFP-encoding HIV-1 vectors, normalized as in (A). (D) Data from (C) but with infectivity plotted against drug concentration divided by affinity to hexamer as calculated by ITC. (E) Titres of VSV-G pseudotyped GFP-encoding HIV-1 vectors 48 h post-infection and levels of reverse transcription (RT) 4 h post-infection under conditions of PF74 inhibition. For both measures, data are normalized to the values obtained in the absence of inhibitor. (F) Data from (E), except plotted against the calculated level of CA occupancy by PF74. The occupancy was calculated assuming that there are 1500 free sites per capsid and that number of free sites = 1500(1−([PF74]/Kd[PF74])) (G) Infectivity and reverse transcription in cells infected in the presence of PF74 (wash) or when removed after four hours (wash out). Reverse transcription was then measured after a further 4 h, while infectivity was determined 48 h post-infection. (H) Levels of reverse transcription 4 h post-infection of HeLa cells in the presence of PF74 and its derivatives at 20× IC9o drug concentrations.

The PF74 derivatives described above also provided an opportunity to test the correlation between compound affinity and potency in infection experiments. As noted earlier, there is a discrepancy in the literature between the published affinity of PF74 and its potency and also between the potency of PF74 and BI-2. When tested, all PF74 derivatives with weaker affinity to hexamer had a correspondingly weaker ability to inhibit HIV-1 infection (Figure 9A). Plotting the IC90 for infection against compound affinity gives a Pearson correlation coefficient of 0.9928 and a P-value of 0.0007, demonstrating that the relationship between affinity to hexamer and potency is highly significant (Figure 9B). These results also suggest that a different affinity for capsid is the reason why PF74 is a more potent inhibitor than BI-2 (Figure 9C). Indeed, when data are normalized using our measurements for drug affinity to hexamer it is apparent that the higher affinity of PF74 completely accounts for the difference between the compounds (Figure 9D).

Finally, we investigated whether our structural and biophysical data could also explain the different phenotypic effects of PF74 and BI-2 on infection. PF74 has a curious inhibition profile in which there is an initial dose-dependent decline in infection, followed by a plateau over a ∼10-fold concentration range then a second inhibition curve (Figure 9C). Parallel infection and qPCR experiments reveal that the first inhibition, at lower drug concentrations, is not associated with any block to viral reverse transcription (Figure 9E). In contrast, the second inhibition, at high drug concentrations, occurs in parallel with a dose-dependent inhibition of reverse transcription. When the data are re-plotted taking into account the measured affinity of PF74 for hexameric CA and expressed as the predicted fraction of unoccupied sites per whole viral capsid, the second block to infection can be seen to occur only at drug concentrations where all six sites in a majority of hexamers become occupied (<5o free sites per capsid) (Figure 9F).

One mechanism by which PF74 might affect reverse transcription at high occupancy is by altering capsid stability. PF74 has been reported to destabilize the capsid [29] and our structures suggest a mechanism by which this could occur, namely by inducing changes at the CTD-CTD interface around helices 8 and 9 that hold hexamers together. To test this hypothesis we examined whether PF74 inhibition is reversible. In wash-out experiments we observed that inhibition of infection by concentrations of PF74 within the first (1 µM) and second (10 or 30 µM) block could be completely and partially reversed, respectively; however, the block to reverse transcription that PF74 mediates at high concentrations was largely irreversible (Figure 9G). To determine whether there is a time window post-infection during which PF74 must be added for efficacy, we performed a time-of-addition experiment. We found that at both low and high doses PF74 quickly lost inhibition if addition was delayed, such that by 10 hours post-infection almost all efficacy was lost (Supplementary Figure S5A). This is further evidence that PF74 acts at a post-entry but pre-integration stage of viral infection. To determine whether the reversibility of PF74 inhibition is affected by the time of drug addition, we repeated our washout experiments but adding PF74 2 hours post-infection rather than coincident with virus. Levels of inhibition were decreased when drug addition was delayed, as expected, but the pattern of reversibility stayed the same (Supplementary Figure S5B&C). Namely, low dose PF74 inhibition was reversed upon washout but high dose inhibition was not. Taken together, these data fit a model in which PF74 inhibits HIV-1 infection at low dose by competing for cofactor binding and at high dose by inducing an irreversible block to reverse transcription, possibly as a result of irreversible changes to the capsid ultrastructure. This model agrees with published data in which the potency of PF74 at low doses is dependent upon the expression of cofactors in the cell and on capsid. Depletion of TNPO3 or NUP153 reduces PF74 inhibition at low but not high doses, as do CA mutants such as N74D or the expression of TRIM-NUP153 [20], [37]. The fact that the low dose PF74 block to infection is not cumulative with the inhibition arising from perturbing these cofactors is consistent with it acting as a competitive inhibitor. Unlike PF74, BI-2 has not been observed to block reverse transcription [28]. Based on the above data, this could either be due to the fact that insufficiently high drug concentrations have been tested to achieve complete occupancy or because it has a different mode of binding. Unfortunately, BI-2 is not soluble at concentrations that would allow this to be tested. However, the weaker affinity of BI-2 for hexamer is not itself a bar to inhibiting reverse transcription, as PF74 derivatives that have weaker affinities than BI-2 block reverse transcription if added at sufficient concentration (Figure 9H).

## Discussion

Four ligands for HIV-1 – two antiviral drugs and two host cofactors – have been shown to bind a shared binding site in monomeric CA. Here we show that this binding site is actually part of a larger protein-protein interface that only exists in the context of hexameric CA. Identification of this interface has revealed that, while there are common interactions principally involving CA N57, there are also ligand-specific contacts. An example of such a contact is N74, which our results show is required for binding to CPSF6 and not NUP153. Other CA residues that are important for binding specifically to CPSF6 include A77, S102, T108, Q179 and K182. Of these, we have shown that S102D and K182R escape restriction by CPSF6ΔNLS, are insensitive to TNPO3 depletion and sensitive to TRIM-NUP153. Conversely, NUP153 makes specific CA interactions that are not used by CPSF6, including with P38, S41, Q63 and R143. In agreement with this, we have identified R143A as a capsid mutant that is susceptible to TNPO3 depletion, sensitive to restriction by CPSF6ΔNLS and insensitive to TRIM-NUP153.

Our data support a model in which both CPSF6 and NUP153 are important HIV-1 cofactors. Engagement of CPSF6 is not required for HIV-1 infection in transformed cell lines [2] but is tightly correlated with dependence on the nuclear entry cofactors TNPO3, NUP153 and NUP358 [38]. There are as yet no examples where perturbing the recruitment of these nuclear entry cofactors (through depletion or CA mutation) reduces infection of viruses that cannot engage CPSF6. Thus, interaction of the capsid with CPSF6 commits HIV-1 to the use of active nuclear pores (as defined by cofactor sensitivity). An important question is therefore why the virus utilizes this pathway if it is not required for infection. However, there are significant consequences for viruses that fail to engage with nuclear entry cofactors. Infection of primary monocyte-derived macrophages depleted of CPSF6 reveals HIV-1 to innate immune sensors, leading to induction of an antiviral state and abrogation of HIV-1 replication [3]. In cells in which innate immune sensing mechanisms are not activated, viruses that are independent of nuclear entry cofactors have altered HIV-1 integration site targeting [9], [11]. Moreover, HIV-1 appears to be under selective pressure *in vivo* to retain CPSF6 binding [38]. It is noteworthy that CPSF6 and NUP153 not only appear to function in the same nuclear import pathway but also share the same CA binding site. This is reminiscent of CypA and NUP358, which similarly compete for binding [16]. Whether this reflects a sequential pathway, with CypA and CPSF6 operating upstream of NUP358 and NUP153, and what the purpose of this might be, remains unclear. Alternatively, we cannot rule out the possibility that CPSF6 engages HIV-1 during passage through the nuclear pore, after NUP153.

The structures presented here allow a protein interaction role to be ascribed to many of the CA mutants that have formed the basis for dissecting HIV-1 post-entry behaviour. However, several CA residues remain for which we cannot currently assign a role in cofactor binding but that nevertheless display specific post-entry defects. Examples of this include E45 and R132, which are located in relative proximity at the top of helices 2 and 7 respectively, between the CPSF6/NUP153 and CypA binding sites. Mutation of R132 results in reduced reverse transcription kinetics [13] and TNPO3 dependence [19] and, in the case of E45A, partial resistance to PF74 [37]. These mutations may have a direct effect on intrinsic capsid stability but they may also disrupt the consequences of cofactor binding in an allosteric manner or alter interactions with as yet unidentified cofactors. CPSF6 and NUP153 and the antiviral drug PF74 all bind an interface that includes helices 8 and 9, previously shown to be flexible in both capsid crystal structures and NMR studies [31], [34]. The flexibility in this region may represent a requirement for capsid to be dynamic but it may also make the interface harder to target by restriction factors, analogous to the ‘conformational masking’ employed by gp120 to avoid neutralizing antibodies [39]. Our data suggest that ligand binding is only possible when this region adopts an “open” conformation. The consequences of fixing this conformation in the bound state may involve changes in capsid structure up to and including uncoating. Indeed, this may explain why at saturating doses of PF74 an irreversible block to infection is observed.

## Materials and Methods

### Production of hexamer, peptides and drugs

HIV-1 CA hexameric proteins, stabilized by engineered inter-subunit disulfide bonds, were produced by assembly of recombinant CA containing four amino acid substitutions, as previously reported [33]. Peptides were synthesized by Designer Bioscience (Cambridge, UK). BI-2 was provided by the Vanderbilt Institute of Chemical Biology Synthesis Core, Vanderbilt University, Nashville TN 37232-0412.

### Synthesis of PF74 and derivatives

Compounds were synthesized using an amine intermediate as previously described [21]. Reactions were monitored by thin layer chromatography (TLC) and final products assessed for purity by NMR and ESI-MS. For full details see Supplementary Text S1.

### Isothermal Titration Calorimetry (ITC)

Hexamer protein was dialysed against ITC buffer (50 mM TRIS pH 7.4, 150 mM NaCl) for experiments using intact hexamer, or ITC buffer containing 1 mM DTT for experiments with monomerized hexamer. The monomeric status of hexamer dialysed against buffer containing DTT was confirmed by size exclusion chromatography. Peptides were dissolved in the same buffer as hexamer; NUP153 peptides were first dissolved in DMSO to a concentration of 8 mM before diluting in buffer. Drugs were dissolved in DMSO to a concentration of 5 mM, before diluting in buffer. In all cases, hexamer (in the syringe) was titrated into the peptide or drug ligand (in the cell), with the exception of the competition experiments, whereby peptide (400 µM) was titrated into hexamer (30 µM) in the presence of drug (100 µM). Typical concentrations used were 600–800 µM hexamer titrated against 60–80 µM peptide unless indicated otherwise, and 200 µM hexamer titrated against 30 µM drug. ITC experiments were conducted on a MicroCal ITC-200, and data analyzed using Origin data analysis software (MicroCal).

### Crystallization, data collection, structure determination and refinement

Crystals were grown at 17°C in sitting well drops. In each case, protein/ligand solution was mixed 1∶1 with reservoir solution, producing crystals within one week to one month. Crystallization conditions were as follows: Hex:CPSF6 crystals were prepared using 0.7 mM hexamer and 4 mM CPSF6 peptide (in 50 mM TRIS pH 8.0) mixed with reservoir solution: (P6) 0.6M sodium potassium tartrate tetrahydrate, 0.1M TRIS pH 8.5; and (P2_1_2_1_2_1_) 20% v/v PEG 300, 10% v/v glycerol, 5% w/v PEG 8K, 0.1 M TRIS pH 8.5. Hex:NUP153 crystals were prepared using 0.4 mM hexamer and 0.6 mM NUP153 peptide (in 50 mM TRIS pH 8.0, 100 mM NaCl) mixed with reservoir solution: (P6) 30% v/v PEG 400, 0.1 M CHES pH 9.5; and (P2_1_2_1_2_1_) 10% w/v PEG 8K, 0.1M imidazole pH 8.0. Hex:PF74 and Hex:BI-2 crystals were prepared by concentrating hexamer with a 2-fold molar excess of drug to a final concentration of 0.4 mM hexamer (in 50 mM TRIS pH 8.0, 100 mM NaCl), before mixing with reservoir solution: (Hex:PF74) 0.2 M magnesium chloride, 8% w/v PEG 20K, 8% v/v PEG 550 MME, 0.1 M TRIS pH 8.5, 3% w/v 1,5-diaminopentane dihydrochloride; and (Hex:BI-2) 12% w/v PEG 4K, 0.1 M TRIS pH 8.5, 3% v/v ethylene glycol. Crystals were cryoprotected with 20% 2-methyl-2-4-pentanediol or 20% ethylene glycol where necessary, before flash-freezing in liquid nitrogen. Data were collected in-house and at Diamond beamlines I04-1 and I24. Crystal data collection and refinement statistics are provided in Table 1. The datasets were processed using the CCP4 program suite [40]. Data were indexed and scaled in MOSFLM and SCALA, respectively. The structure was determined by molecular replacement in PHASER using HIV-1 CA hexamer pdb 3H47 as a model for P6 structures, and pdb 3H4E as a model for P2_1_2_1_2_1_ structures. Structural figures were prepared using PyMOL (MacPyMOL Molecular Graphics System, 2009, DeLano Scientific LLC).

### Cells and viruses

All cell lines were obtained from the American Tissue Culture Collection (ATCC) unless otherwise stated. HeLa and Human Embryonic Kidney 293 cells expressing the SV40 large T antigen (HEK293T) cell lines were maintained in Dulbecco's Modified Eagle Medium (DMEM) supplemented with 10% fetal calf serum, penicillin at 100 U/ml and streptomycin at 100 µg/ml. Replication deficient VSV-G pseudotyped HIV GFP vectors were produced by transfection of HEK293T cells with 1.5 µg HIV-1 Gag-Pol expression plasmid (pCRV-1 [41]), 1.5 µg HIV-GFP encoding plasmid (CSGW [42]) and 1 µg VSV-G glycoprotein expression plasmid (pMD2G [43]) using Fugene-6 (Promega). Supernatant was harvested 3 days post-transfection and passed through a 0.45 µm filter. Mutagenesis of CA was performed using the QuikChange method (Stratagene) against pCRV-1. Viral p24 levels were quantified using Lenti-X p24 Rapid Titer Kit (Clontech).

### Infection experiments

Infections were performed in the presence of 5 µg/ml polybrene and GFP expressing cells were enumerated on a BD LSRII Flow Cytometer (BD Biosciences) 2 days post-transfection after fixation of cells in 4% paraformaldehyde. Where used, drugs dissolved in DMSO or DMSO only were diluted in complete DMEM supplemented with polybrene as above and added to cells shortly before infection. For wash out experiments, cells were washed twice with PBS 4 h post-infection and complete DMEM with DMSO (wash out) or PF74 (wash) was added back to the cells. Stable TNPO3 depletion experiments were performed by transducing HeLa cells (1×10^5^) with retroviral vectors (pSIREN RetroQ) expressing TNPO3-specific short hairpin RNA (shRNA). Cells were selected with 10 µg/ml puromycin and stable cell-lines used in infection experiments. For further details see [9]. For CPSF6ΔNLS restriction experiments, a C-terminally deleted CPSF6 construct (residues 1–504) was cloned from HeLa cDNA (accession number NM_007007) and stably expressed following transduction of HeLa cells with retroviral vector (pEXN). Cells were selected with 1 mg/ml G418. And stable-cell lines used in infection experiments. For further details see [3]. For TRIM-NUP153 restriction experiments, constructs containing an internal HA tag or C-terminal HA tag (as previously published [20]) or N-terminal HA tag were transduced into HeLa cells and selected with puromycin or G418 respectively. All three constructs contained NUP153 residues 896–1475 fused to the C-terminus of the tripartite domains of TRIM5 (as described [20]). A stable cell line expressing the N-terminal tagged construct was then used in infection experiments. In all cases, infection was carried out in 1×10^5^ HeLa cells at a multiplicity of infection (m.o.i.) between 0.1–0.3 using VSV-G pseudotyped HIV GFP vector.

### Quantitative PCR

For analysis of reverse transcription products, viral supernatant was treated with 250 U/ml Benzonase (Millipore) for 20 min prior to infection as above. DNA was extracted using DNeasy Blood and Tissue Kit (Qiagen). GFP copies were quantified using primers GFPF (CAACAGCCACAACGTCTATATCAT), GFPR (ATGTTGTGGCGGATCTTGAAG) and probe GFPP (FAM-CCGACAAGCAGAAGAACGGCATCAA-TAMRA) against a standard curve of CSGW on an ABI StepOnePlus Real Time PCR System (Life Technologies).

### Accession numbers

All structures have been deposited in the PDB. 4U0A: Hex:CPSF6 P6, 4U0B: Hex:CPSF6 P212121, 4U0C: Hex:Nup153 P6, 4U0D: Hex:Nup153 P212121, 4U0E: Hex:PF74 P6, 4U0F: Hex:BI-2 P6.

## Supporting Information

Figure S1 **Superposition of hexamer:ligand complexed structures obtained in different spacegroups.** In each case the P6 and P2_1_2_1_2_1_ hexamer structures in complex with either NUP153 or CPSF6 have been superposed. The P6 structures are shown in gray and P2_1_2_1_2_1_ in yellow. A close-up view of the binding site is shown, comprising two adjacent monomers. The capsid is depicted in a secondary structure representation whilst the ligands are shown in stick form.
(PDF)

Figure S2 **Binding of HIV-1 hexamer to CPSF6 and NUP153 mutant peptides by ITC.** ITC isotherms are shown for the named titrants. The concentration of each titrant is also given. Approximate affinities are shown for interactions where a binding isotherm could be fitted. NB = no binding detectable.
(PDF)

Figure S3 **Water-mediated interactions between NUP153 peptide and HIV-1 hexamer.** A close-up view of the binding site in the P6 complexed structure between HIV-1 hexamer and NUP153 peptide is shown. Parts of two adjacent capsid monomers are shown in gray and teal. NUP153 is shown in pink in a stick representation. Water molecules are indicates as blue spheres and are numbered according to the deposited crystal structure. Important hexamer and peptide residues are labelled, with peptide labels in italic.
(PDF)

Figure S4 **Superposition of PF74 and BI-2 on the uncomplexed P2_1_2_1_2_1_ hexamer structure (pdb 3H4E).** The ‘open’ and ‘closed’ states adopted in 3H4E as shown in Figure 3B, superposed onto the orthorhombic hexamer structures of PF74 and BI-2. A close-up view of the binding site is shown in which one monomer is in gray, while the ‘open’ and ‘closed’ states of the second monomer in 3H4E are shown in teal and light green respectively. PF74 from the superposed complexed structure is shown in green (left) whilst BI-2 is shown in orange (right). As can be seen, PF74, but not BI-2, clashes with the ‘closed’ conformation.
(PDF)

Figure S5 **Reversibility and kinetics of PF74 inhibition.** (A) Time-of-addition experiment in which PF74 was added to HeLa cells at either low (1 µM) or high (30 µM) doses at different times post-infection with VSV-G pseudotyped GFP-encoding HIV-1 vector. Infectivity was then determined after 48 hours and normalized to cells treated with DMSO. (B & C) PF74 was added coincident with infection or 2 hours post-infection. Cells were transferred into media with (wash) or without (washout) PF74 after 4 hours of drug treatment. Reverse transcription was determined after 4 hours of recovery (B), while infection levels were determined after 48 hours (C).
(PDF)

Text S1 **Chemical synthesis of PF74 derivatives.** Synthetic schemes are provided for PF74 compounds. Data from mass spectra (ESI-MS) and NMR spectra are given for each compound.
(DOCX)

## References

[1] Franke EK, Yuan HE, Luban J (1994) Specific incorporation of cyclophilin A into HIV-1 virions. Nature 372: 359–362. 7969494 10.1038/372359a0

[2] Lee K, Ambrose Z, Martin TD, Oztop I, Mulky A, et al (2010) Flexible use of nuclear import pathways by HIV-1. Cell Host Microbe 7: 221–233. 20227665 10.1016/j.chom.2010.02.007 PMC2841689

[3] Rasaiyaah J, Tan CP, Fletcher AJ, Price AJ, Blondeau C, et al (2013) HIV-1 evades innate immune recognition through specific cofactor recruitment. Nature 503: 402–405. 24196705 10.1038/nature12769 PMC3928559

[4] Brass AL, Dykxhoorn DM, Benita Y, Yan N, Engelman A, et al (2008) Identification of host proteins required for HIV infection through a functional genomic screen. Science 319: 921–926. 18187620 10.1126/science.1152725

[5] Bushman FD, Malani N, Fernandes J, D'Orso I, Cagney G, et al (2009) Host cell factors in HIV replication: meta-analysis of genome-wide studies. PLoS Pathog 5: e1000437. 19478882 10.1371/journal.ppat.1000437 PMC2682202

[6] Konig R, Zhou Y, Elleder D, Diamond TL, Bonamy GM, et al (2008) Global analysis of host-pathogen interactions that regulate early-stage HIV-1 replication. Cell 135: 49–60. 18854154 10.1016/j.cell.2008.07.032 PMC2628946

[7] Cherepanov P, Maertens G, Proost P, Devreese B, Van Beeumen J, et al (2003) HIV-1 integrase forms stable tetramers and associates with LEDGF/p75 protein in human cells. The Journal of biological chemistry 278: 372–381. 12407101 10.1074/jbc.M209278200

[8] Towers GJ, Hatziioannou T, Cowan S, Goff SP, Luban J, et al (2003) Cyclophilin A modulates the sensitivity of HIV-1 to host restriction factors. Nat Med 9: 1138–1143. 12897779 10.1038/nm910

[9] Schaller T, Ocwieja KE, Rasaiyaah J, Price AJ, Brady TL, et al (2011) HIV-1 Capsid-Cyclophilin Interactions Determine Nuclear Import Pathway, Integration Targeting and Replication Efficiency. PLoS Pathog 7: e1002439. 22174692 10.1371/journal.ppat.1002439 PMC3234246

[10] Krishnan L, Matreyek KA, Oztop I, Lee K, Tipper CH, et al (2010) The requirement for cellular transportin 3 (TNPO3 or TRN-SR2) during infection maps to human immunodeficiency virus type 1 capsid and not integrase. J Virol 84: 397–406. 19846519 10.1128/JVI.01899-09 PMC2798409

[11] Ocwieja KE, Brady TL, Ronen K, Huegel A, Roth SL, et al (2011) HIV Integration Targeting: A Pathway Involving Transportin-3 and the Nuclear Pore Protein RanBP2. PLoS Pathog 7: e1001313. 21423673 10.1371/journal.ppat.1001313 PMC3053352

[12] Christ F, Thys W, De Rijck J, Gijsbers R, Albanese A, et al (2008) Transportin-SR2 imports HIV into the nucleus. Curr Biol 18: 1192–1202. 18722123 10.1016/j.cub.2008.07.079

[13] Forshey BM, von Schwedler U, Sundquist WI, Aiken C (2002) Formation of a human immunodeficiency virus type 1 core of optimal stability is crucial for viral replication. J Virol 76: 5667–5677. 11991995 10.1128/JVI.76.11.5667-5677.2002 PMC137032

[14] Hulme AE, Perez O, Hope TJ (2011) Complementary assays reveal a relationship between HIV-1 uncoating and reverse transcription. Proc Natl Acad Sci U S A 108: 9975–9980. 21628558 10.1073/pnas.1014522108 PMC3116424

[15] Yamashita M, Emerman M (2004) Capsid is a dominant determinant of retrovirus infectivity in nondividing cells. J Virol 78: 5670–5678. 15140964 10.1128/JVI.78.11.5670-5678.2004 PMC415837

[16] Bichel K, Price AJ, Schaller T, Towers GJ, Freund SM, et al (2013) HIV-1 capsid undergoes coupled binding and isomerization by the nuclear pore protein NUP358. Retrovirology 10: 81. 23902822 10.1186/1742-4690-10-81 PMC3750474

[17] Zhao Y, Chen Y, Schutkowski M, Fischer G, Ke H (1997) Cyclophilin A complexed with a fragment of HIV-1 gag protein: insights into HIV-1 infectious activity. Structure 5: 139–146. 9016720 10.1016/s0969-2126(97)00172-x

[18] Braaten D, Luban J (2001) Cyclophilin A regulates HIV-1 infectivity, as demonstrated by gene targeting in human T cells. Embo J 20: 1300–1309. 11250896 10.1093/emboj/20.6.1300 PMC145517

[19] De Iaco A, Luban J (2011) Inhibition of HIV-1 infection by TNPO3 depletion is determined by capsid and detectable after viral cDNA enters the nucleus. Retrovirology 8: 98. 22145813 10.1186/1742-4690-8-98 PMC3267670

[20] Matreyek KA, Yucel SS, Li X, Engelman A (2013) Nucleoporin NUP153 phenylalanine-glycine motifs engage a common binding pocket within the HIV-1 capsid protein to mediate lentiviral infectivity. PLoS pathogens 9: e1003693. 24130490 10.1371/journal.ppat.1003693 PMC3795039

[21] Price AJ, Fletcher AJ, Schaller T, Elliott T, Lee K, et al (2012) CPSF6 defines a conserved capsid interface that modulates HIV-1 replication. PLoS pathogens 8: e1002896. 22956906 10.1371/journal.ppat.1002896 PMC3431306

[22] Matreyek KA, Engelman A (2011) The requirement for nucleoporin NUP153 during human immunodeficiency virus type 1 infection is determined by the viral capsid. J Virol 85: 7818–7827. 21593146 10.1128/JVI.00325-11 PMC3147902

[23] Ambrose Z, Aiken C (2014) HIV-1 uncoating: connection to nuclear entry and regulation by host proteins. Virology 454–455C: 371–379. 10.1016/j.virol.2014.02.004 PMC3988234 24559861

[24] Maertens GN, Cook NJ, Wang W, Hare S, Gupta SS, et al (2014) Structural basis for nuclear import of splicing factors by human Transportin 3. Proceedings of the National Academy of Sciences of the United States of America 111: 2728–2733. 24449914 10.1073/pnas.1320755111 PMC3932936

[25] De Iaco A, Santoni F, Vannier A, Guipponi M, Antonarakis S, et al (2013) TNPO3 protects HIV-1 replication from CPSF6-mediated capsid stabilization in the host cell cytoplasm. Retrovirology 10: 20. 23414560 10.1186/1742-4690-10-20 PMC3599327

[26] Yamashita M, Perez O, Hope TJ, Emerman M (2007) Evidence for direct involvement of the capsid protein in HIV infection of nondividing cells. PLoS Pathog 3: 1502–1510. 17967060 10.1371/journal.ppat.0030156 PMC2042020

[27] Blair WS, Pickford C, Irving SL, Brown DG, Anderson M, et al (2010) HIV capsid is a tractable target for small molecule therapeutic intervention. PLoS Pathog 6: e1001220. 21170360 10.1371/journal.ppat.1001220 PMC3000358

[28] Lamorte L, Titolo S, Lemke CT, Goudreau N, Mercier JF, et al (2013) Discovery of Novel Small-Molecule HIV-1 Replication Inhibitors That Stabilize Capsid Complexes. Antimicrobial agents and chemotherapy 57: 4622–4631. 23817385 10.1128/AAC.00985-13 PMC3811413

[29] Shi J, Zhou J, Shah VB, Aiken C, Whitby K (2011) Small-molecule inhibition of human immunodeficiency virus type 1 infection by virus capsid destabilization. J Virol 85: 542–549. 20962083 10.1128/JVI.01406-10 PMC3014163

[30] Hori T, Takeuchi H, Saito H, Sakuma R, Inagaki Y, et al (2013) A carboxy-terminally truncated human CPSF6 lacking residues encoded by exon 6 inhibits HIV-1 cDNA synthesis and promotes capsid disassembly. Journal of virology 87: 7726–7736. 23658440 10.1128/JVI.00124-13 PMC3700264

[31] Pornillos O, Ganser-Pornillos BK, Kelly BN, Hua Y, Whitby FG, et al (2009) X-ray structures of the hexameric building block of the HIV capsid. Cell 137: 1282–1292. 19523676 10.1016/j.cell.2009.04.063 PMC2840706

[32] Fricke T, Brandariz-Nunez A, Wang X, Smith AB 3rd, Diaz-Griffero F (2013) Human cytosolic extracts stabilize the HIV-1 core. Journal of virology 87: 10587–10597. 23885082 10.1128/JVI.01705-13 PMC3807412

[33] Pornillos O, Ganser-Pornillos BK, Banumathi S, Hua Y, Yeager M (2010) Disulfide bond stabilization of the hexameric capsomer of human immunodeficiency virus. J Mol Biol 401: 985–995. 20600115 10.1016/j.jmb.2010.06.042 PMC3050670

[34] Byeon IJ, Hou G, Han Y, Suiter CL, Ahn J, et al (2012) Motions on the millisecond time scale and multiple conformations of HIV-1 capsid protein: implications for structural polymorphism of CA assemblies. Journal of the American Chemical Society 134: 6455–6466. 22428579 10.1021/ja300937v PMC3325613

[35] Lee K, Mulky A, Yuen W, Martin TD, Meyerson NR, et al (2012) HIV-1 Capsid-Targeting Domain of Cleavage and Polyadenylation Specificity Factor 6. J Virol 86: 3851–3860. 22301135 10.1128/JVI.06607-11 PMC3302544

[36] Gallivan JP, Dougherty DA (2000) A Computational Study of Cation-Pi Interactions vs Salt Bridges in Aqueous Media: Implications for Protein Engineering. J Am Chem Soc 122: 870–874.

[37] Shah VB, Shi J, Hout DR, Oztop I, Krishnan L, et al (2013) The host proteins transportin SR2/TNPO3 and cyclophilin A exert opposing effects on HIV-1 uncoating. Journal of virology 87: 422–432. 23097435 10.1128/JVI.07177-11 PMC3536424

[38] Henning MS, Dubose BN, Burse MJ, Aiken C, Yamashita M (2014) In vivo functions of CPSF6 for HIV-1 as revealed by HIV-1 capsid evolution in HLA-B27-positive subjects. PLoS pathogens 10: e1003868. 24415937 10.1371/journal.ppat.1003868 PMC3887095

[39] Kwong PD, Doyle ML, Casper DJ, Cicala C, Leavitt SA, et al (2002) HIV-1 evades antibody-mediated neutralization through conformational masking of receptor-binding sites. Nature 420: 678–682. 12478295 10.1038/nature01188

[40] Collaborative Computational Project N (1994) The CCP4 suite: programs for protein crystallography. Acta Crystallogr D Biol Crystallogr 50: 760–763. 15299374 10.1107/S0907444994003112

[41] Zennou V, Perez-Caballero D, Gottlinger H, Bieniasz PD (2004) APOBEC3G incorporation into human immunodeficiency virus type 1 particles. Journal of virology 78: 12058–12061. 15479846 10.1128/JVI.78.21.12058-12061.2004 PMC523273

[42] Naldini L, Blomer U, Gallay P, Ory D, Mulligan R, et al (1996) In vivo gene delivery and stable transduction of nondividing cells by a lentiviral vector. Science 272: 263–267. 8602510 10.1126/science.272.5259.263

[43] Yee JK, Friedmann T, Burns JC (1994) Generation of high-titer pseudotyped retroviral vectors with very broad host range. Methods in cell biology 43 Pt A: 99–112. 7823872 10.1016/s0091-679x(08)60600-7

[44] Krissinel E, Henrick K (2007) Inference of macromolecular assemblies from crystalline state. Journal of molecular biology 372: 774–797. 17681537 10.1016/j.jmb.2007.05.022

[45] Laskowski RA, Swindells MB (2011) LigPlot+: multiple ligand-protein interaction diagrams for drug discovery. Journal of chemical information and modeling 51: 2778–2786. 21919503 10.1021/ci200227u

