# Supplementary figures and images for: Host Cofactors and Pharmacologic Ligands Share an Essential Interface in HIV-1 Capsid That Is Lost upon Disassembly
Source: PLoS Pathog. 2014 Oct 30;10(10):e1004459. doi: 10.1371/journal.ppat.1004459 (PMC4214760; doi:10.1371/journal.ppat.1004459)

# Supplementary Figure 1

Nup153

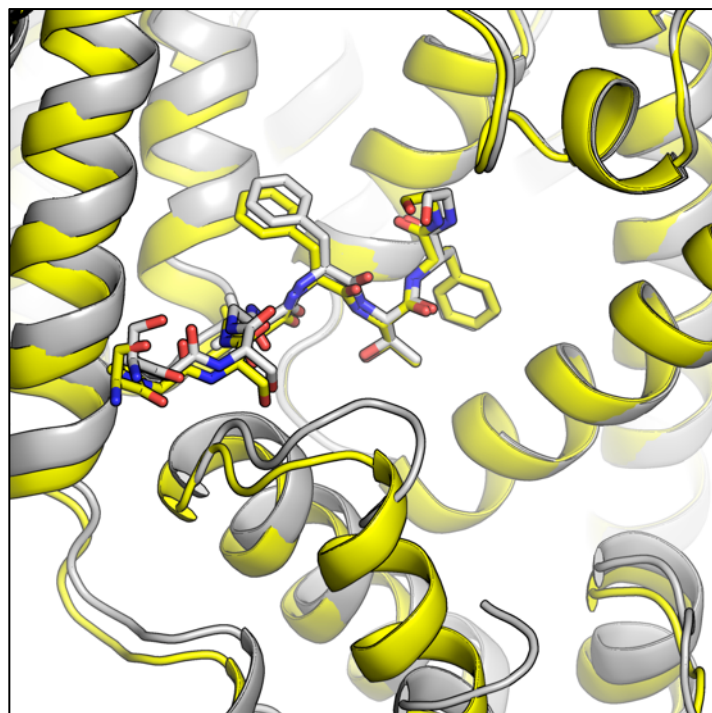

CPSF6

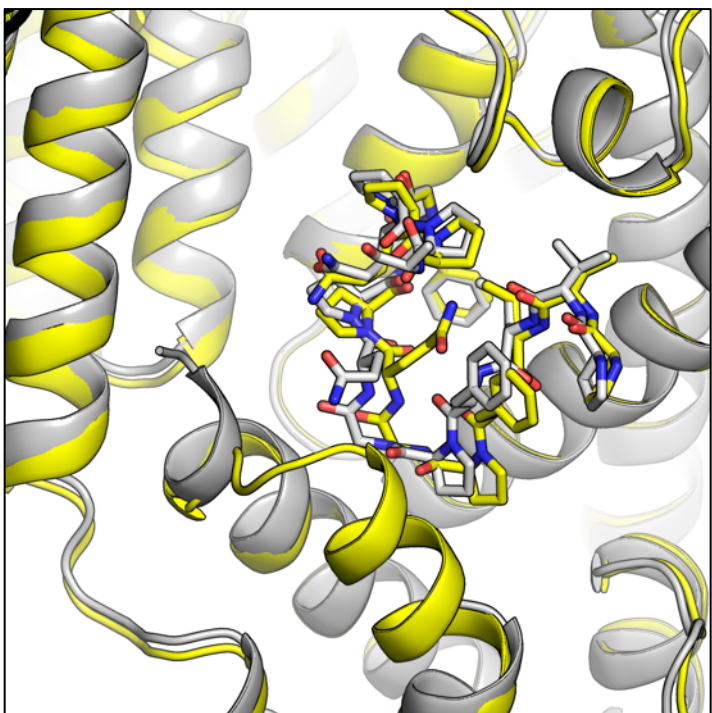

Supplement: Figure S1 — Superposition of hexamer:ligand complexed structures obtained in different spacegroups. In each case the P6 and P212121 hexamer structures in complex with either NUP153 or CPSF6 have been superposed. The P6 structures are shown in gray and P212121 in yellow. A close-up view of the binding site is shown, comprising two adjacent monomers. The capsid is depicted in a secondary structure representation whilst the ligands are shown in stick form. (PDF) [file ppat.1004459.s001.pdf]

# Supplementary Figure 2

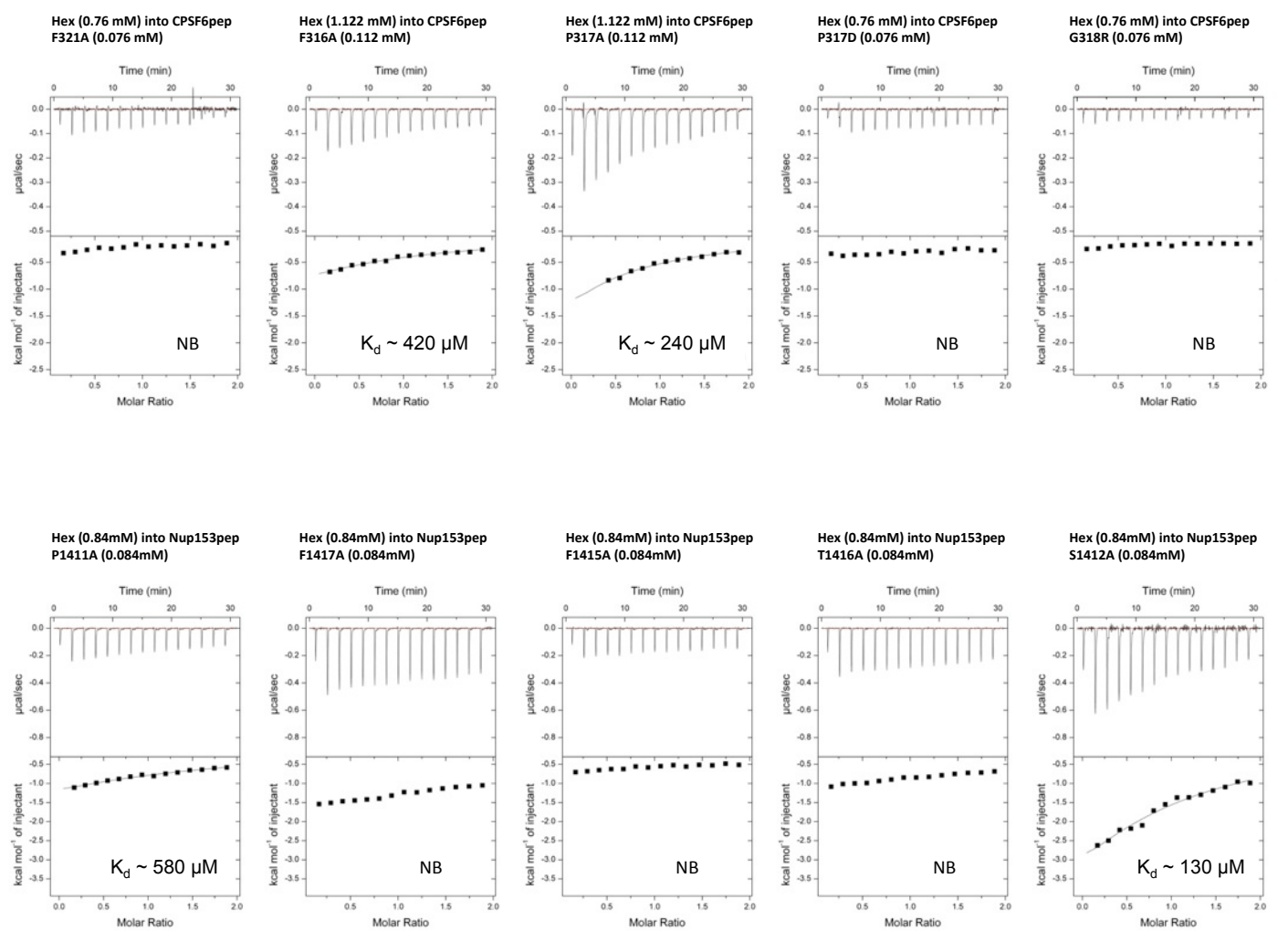

Supplement: Figure S2 — Binding of HIV-1 hexamer to CPSF6 and NUP153 mutant peptides by ITC. ITC isotherms are shown for the named titrants. The concentration of each titrant is also given. Approximate affinities are shown for interactions where a binding isotherm could be fitted. NB = no binding detectable. (PDF) [file ppat.1004459.s002.pdf]

Supplementary Figure 3

Nup153

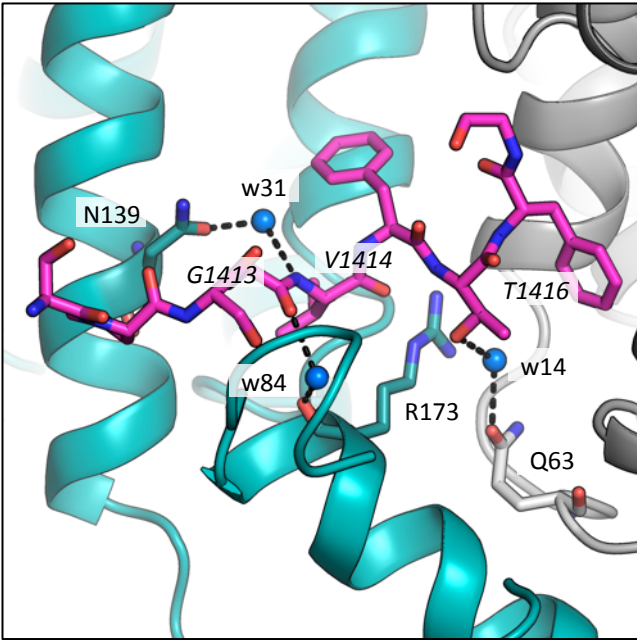

Supplement: Figure S3 — Water-mediated interactions between NUP153 peptide and HIV-1 hexamer. A close-up view of the binding site in the P6 complexed structure between HIV-1 hexamer and NUP153 peptide is shown. Parts of two adjacent capsid monomers are shown in gray and teal. NUP153 is shown in pink in a stick representation. Water molecules are indicates as blue spheres and are numbered according to the deposited crystal structure. Important hexamer and peptide residues are labelled, with peptide labels in italic. (PDF) [file ppat.1004459.s003.pdf]

Supplementary Figure 4

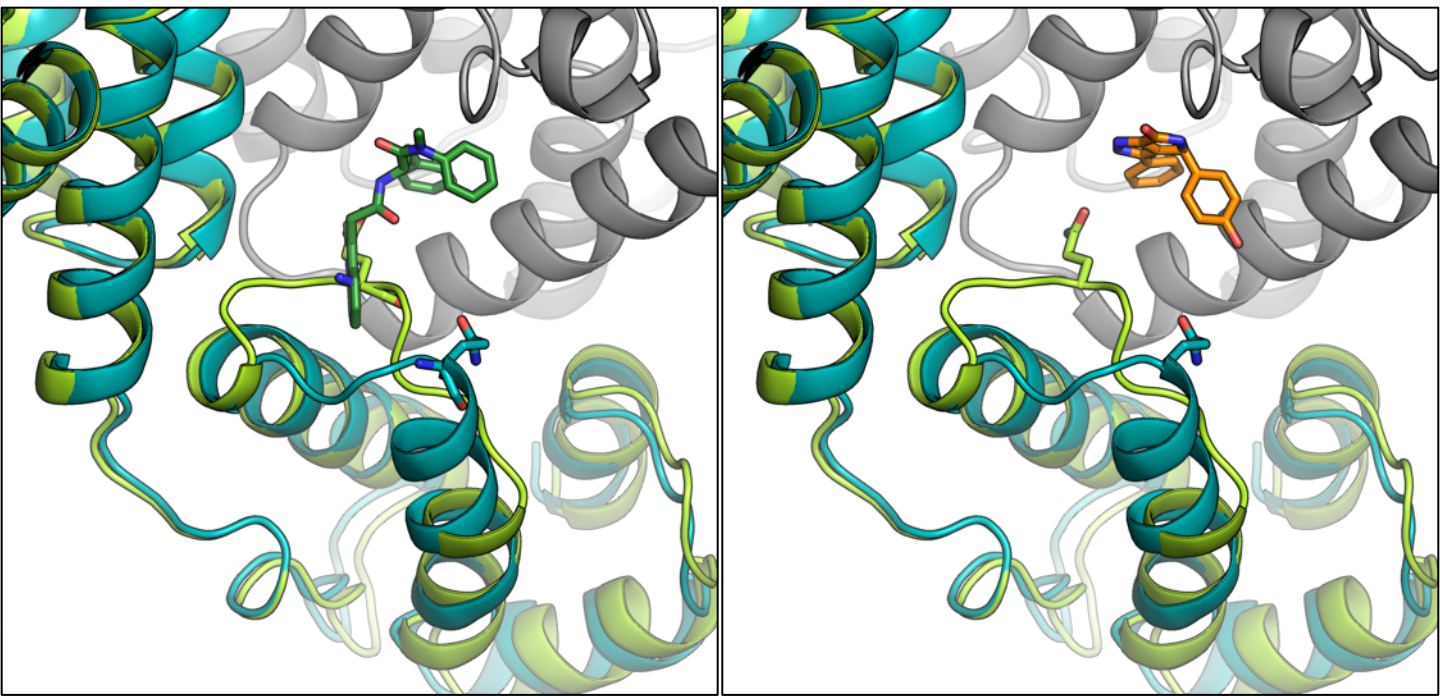

Supplement: Figure S4 — Superposition of PF74 and BI-2 on the uncomplexed P212121 hexamer structure (pdb 3H4E). The ‘open’ and ‘closed’ states adopted in 3H4E as shown in Figure 3B, superposed onto the orthorhombic hexamer structures of PF74 and BI-2. A close-up view of the binding site is shown in which one monomer is in gray, while the ‘open’ and ‘closed’ states of the second monomer in 3H4E are shown in teal and light green respectively. PF74 from the superposed complexed structure is shown in green (left) whilst BI-2 is shown in orange (right). As can be seen, PF74, but not BI-2, clashes with the ‘closed’ conformation. (PDF) [file ppat.1004459.s004.pdf]

# Supplementary Figure 5

A

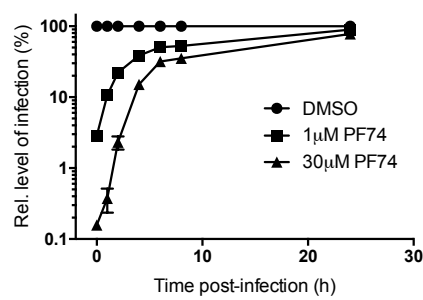

B

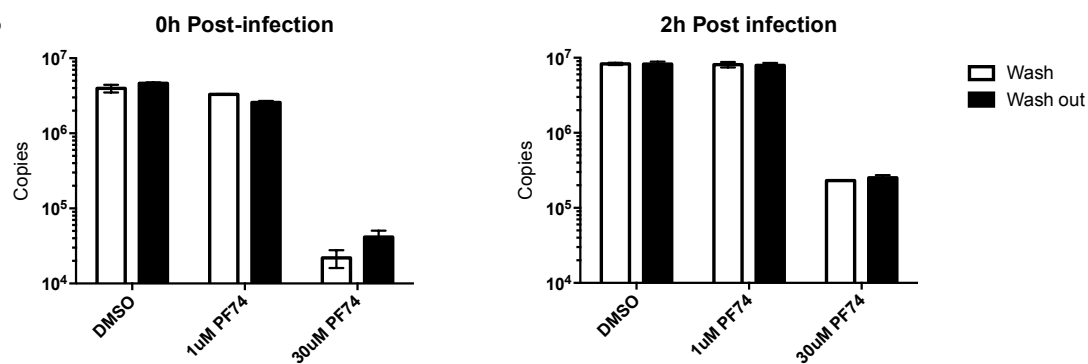

C

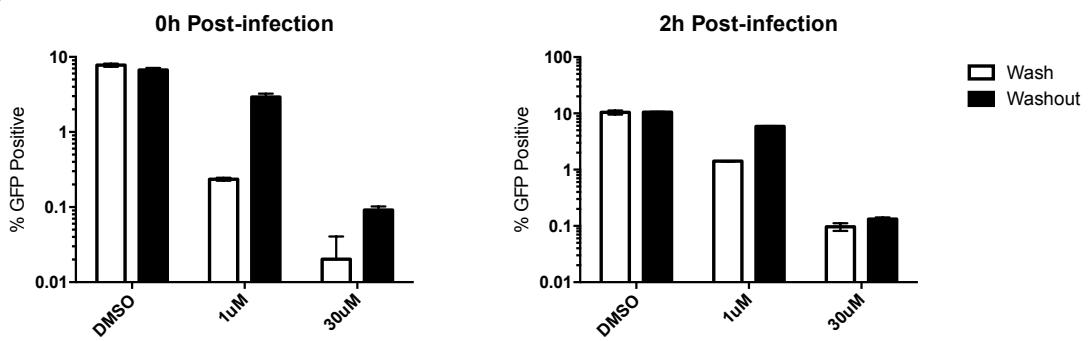

Supplement: Figure S5 — Reversibility and kinetics of PF74 inhibition. (A) Time-of-addition experiment in which PF74 was added to HeLa cells at either low (1 µM) or high (30 µM) doses at different times post-infection with VSV-G pseudotyped GFP-encoding HIV-1 vector. Infectivity was then determined after 48 hours and normalized to cells treated with DMSO. (B & C) PF74 was added coincident with infection or 2 hours post-infection. Cells were transferred into media with (wash) or without (washout) PF74 after 4 hours of drug treatment. Reverse transcription was determined after 4 hours of recovery (B), while infection levels were determined after 48 hours (C). (PDF) [file ppat.1004459.s005.pdf]
