# Supplementary material for: Host Cofactors and Pharmacologic Ligands Share an Essential Interface in HIV-1 Capsid That Is Lost upon Disassembly
Source: PLoS Pathog. 2014 Oct 30;10(10):e1004459. doi: 10.1371/journal.ppat.1004459 (PMC4214760; doi:10.1371/journal.ppat.1004459)
Supplement: Text S1 — Chemical synthesis of PF74 derivatives. Synthetic schemes are provided for PF74 compounds. Data from mass spectra (ESI-MS) and NMR spectra are given for each compound. (DOCX) [file ppat.1004459.s006.docx]

**Chemical Synthesis**

**General Remarks**

**Solvents and Reagents**: Unless stated otherwise, solvents and dry solvents were purchased from Sigma-Aldrich. Unless stated otherwise all of these chemicals were used without further purification. Amine **2** was synthesized like previously described.^15^

**Reaction Handling:** Unless stated otherwise all non-aqueous reactions were performed in flame-dried glassware under an atmosphere of argon. All flasks were equipped with rubber septa and reactants were handled using standard Schlenk techniques. Temperatures above the room temperature (23°C) refer to oil bath temperatures which were controlled by a temperature modulator. Reactions were magnetically stirred and monitored by thin layer chromatography **(**TLC) unless otherwise noted.

**Analytical Thin Layer Chromatography** was carried out with "LuxPlate^®^ silica gel 60 F254" sheets from Merck. Detection was carried out using UV light (254 nm and 366 nm) or permanganate (0.6% KMnO_4_ in water with 1.0% K_2_CO_3_). Concentrations under reduced pressure were performed by rotary evaporation at 40°C at the appropriated pressure, unless otherwise noted. **Flash column chromatography** was accomplished using silica gel S (pore size 60 Å, 0.040-0.063 mm) from Sigma-Aldrich. The yields given refer to the isolated yields.

**^1^H-NMR** spectra were recorded at room temperature on Bruker AVB-400 spectrometer with ^1^H operating frequency of 400 MHz. Unless stated otherwise all spectra were recorded at room temperature in CDCl_3_ and all chemical shifts are given in δ units relative to CHCl_3_ (central line of singlet: δH = 7.27). Analyses followed first order and the following abbreviations were used throughout: s = singlet, br. s. = broad singlet, d = doublet, t = triplet, q = quartet, quin = quintet, sxt = sextet, sept = spt, dd = doublet of doublet, dt = doublet of triplet, m = multiplet, m_c_ = centered multiplet. Coupling constants through n bonds (^n^*J*) are given in Hertz [Hz].

**^13^C-NMR** spectra were recorded at room temperature on Bruker AVB-400 spectrometer with ^13^C operating frequency of 101 MHz. Unless stated otherwise all spectra were recorded at room temperature in CDCl_3_ and all chemical shifts are given in δ units relative to CHCl_3_ (central line of triplet: δC = 77.00). The following abbreviation was used throughout: s = singlet, d = doublet, dd = doublet of doublet. If no coupling constants are given, the multiplicity refers to the ^1^H-decoupled spectra.

^a^ chem. shifts associated with diastereomer a are marked with a.

^b^ chem. shifts associated with diastereomer b are marked with b.

**Mass spectra (MS)** were recorded on an Agilent 1200 LC-MS system with a 6130 Quadrupole spectrometer for ESI-MS. The solvent system consisted of 0.2 % formic acid in H_2_O as buffer A, and 0.2 % formic acid in MeCN as buffer B. Small molecule LC-MS was carried out using a Phenomenex Jupiter C18 column (150 x 2 mm, 5 μm). Variable wavelengths were used and MS acquisitions were carried out in positive and negative ion modes.

**Scheme.** Synthesis of the PF74 derivatives PF74-3 to PF74-6.

Representative procedure for the amide coupling with **2**:

Amine **2** (150 mg, 589 µmol, 1.00 eq), 2-(1H-pyrrol-3-yl)acetic acid (88.6 mg, 707 µmol, 1.20 eq), EDCI hydrochloride (192 mg, 1.00 mmol, 1.70 eq) and BtOH (13.5 mg, 88.4 µmol, 0.15 eq) were dissolved in dry DMF (4.0 mL) and were stirred for 16 h at room temperature. Subsequently, water (15 mL) was added and the mixture was extracted with 3 x 20 mL EtOAc, dried over MgSO_4_ and filtered off. The solvent was then evaporated under reduced pressure at 60°C and the crude product was purified by flash column chromatography on silica gel (hexane/ethyl acetate 1:1 to pure ethyl acetate) to afford the corresponding PF74 derivative PF74-3 (**3**) (166 mg, 459 µmol, 78%).

**TLC**: R*_f_* = 0.21 (hexane/ethyl acetate 1:1). **^1^H NMR** (CDCl_3_, 400 MHz): δ = 2.66 (1 H, dd, *J* = 13.4, 7.4 Hz), 2.87 (1 H, dd, *J* = 13.5, 6.9 Hz), 3.23 (3 H, s), 3.50 (2 H, s), 4.78 (1 H, m_C_), 5.92 - 6.02 (1 H, m), 6.14 (1 H, dd, *J* = 5.5, 2.5 Hz), 6.31 (1 H, d, *J* = 7.9 Hz), 6.66 - 6.75 (1 H, m), 6.79 - 6.86 (2 H, m), 6.90 - 7.00 (2 H, m), 7.13 - 7.24 (3 H, m), 7.32 - 7.41 (3 H, m), 8.71 (1 H, br. s.). **^13^C NMR** (CDCl_3_, 101 MHz): δ = 35.3, 37.7, 38.8, 51.3, 107.5, 108.4, 117.9, 124.2, 126.9, 127.3 (2 C), 128.2, 128.4 (2 C), 129.2 (2 C), 129.8 (2 C), 136.1, 142.4, 169.8, 171.3. **ESI-MS** [M+H]^+^ calculated for C_22_H_24_O_3_N_3_: 362.4; found: 362.3.

The representative procedure given for PF74 derivative PF74-3 (**3**) was carried out with 2-(furan-3-yl)acetic acid (89 mg, 707 µmol, 1.20 eq) to afford PF74-4 (**4**) (171 mg, 471 µmol, 80% yield) after flash column chromatography on silica gel (hexane/ethyl acetate 1:1 to pure ethyl acetate).

**TLC**: R*_f_* = 0.23 (hexane/ethyl acetate 1:1). **^1^H NMR** (CDCl_3_, 400 MHz): δ = 2.67 (1 H, dd, *J =* 13.3, 7.2 Hz), 2.87 (1 H, dd, *J =* 13.3, 7.2 Hz), 3.22 (3 H, s), 3.33 (2 H, d, *J =* 5.9 Hz), 4.82 (1 H, m_C_), 6.22 - 6.28 (2 H, m), 6.85 (2 H, dd, J = 7.2, 2.0 Hz), 6.89 - 6.96 (2 H, m), 7.16 - 7.23 (3 H, m), 7.30 - 7.32 (1 H, m), 7.33 - 7.38 (3 H, m), 7.40 (1 H, dd, *J =* 1.7, 1.5 Hz). **^13^C NMR** (CDCl_3_, 101 MHz): δ = 32.8, 37.6, 38.9, 51.1, 111.2, 117.9, 126.9, 127.3 (2 C), 128.2, 128.4 (2 C), 129.2 (2 C), 129.8 (2 C), 136.1, 140.7, 142.4, 143.5, 169.6, 171.3. **ESI-MS** [M+H]^+^ calculated for C_22_H_23_O_3_N_2_: 363.4; found: 363.2.

The representative procedure given for PF74 derivative PF74-3 (**3**) was carried out with 3-thiopheneacetic acid (400 mg, 977 µmol, 1.20 eq) to afford PF74-5 (**5**) (171 mg, 452 µmol, 77% yield) after flash column chromatography on silica gel (hexane/ethyl acetate 1:1 to pure ethyl acetate).

**TLC**: R*_f_* = 0.25 (hexane/ethyl acetate 1:1). **^1^H NMR** (CDCl_3_, 400 MHz): δ = 2.65 (1 H, dd, *J =* 13.3, 7.2 Hz), 2.85 (1 H, dd, *J =* 13.3, 6.9 Hz), 3.22 (3 H, s), 3.53 (2 H, d, *J =* 6.0 Hz), 4.74 - 4.88 (1 H, m), 6.09 - 6.26 (1 H, m), 6.81 (2 H, dd, *J =* 7.4, 1.7 Hz), 6.88 - 6.92 (1 H, m), 6.92 - 6.97 (2 H, m), 7.04 - 7.07 (1 H, m), 7.15 - 7.23 (3 H, m), 7.30 (1 H, dd, *J =* 4.8, 2.9 Hz), 7.33 - 7.39 (3 H, m). **^13^C NMR** (CDCl_3_, 101 MHz): δ = 37.6, 37.9, 38.8, 51.1, 123.2, 126.4, 126.8, 127.3 (2 C), 128.1, 128.4 (2 C), 128.4, 129.2 (2 C), 129.8 (2 C), 134.5, 136.1, 142.4, 169.7, 171.3. **ESI-MS** [M+H]^+^ calculated for C_22_H_23_O_2_N_2_S: 379.5; found: 379.2.

The representative procedure given for PF74 derivative PF74-3 (**3**) was carried out with (tetrahydrofuran-3-yl)acetic acid (92 mg, 707 µmol, 1.20 eq) to afford PF74-6 (**6**) (157 mg, 433 µmol, 74% yield) after flash column chromatography on silica gel (hexane/ethyl acetate 3:1 to hexane/ethyl acetate 1:1).

**TLC**: R*_f_* = 0.17 (hexane/ethyl acetate 3:1). **^1^H NMR** (CDCl_3_, 400 MHz): δ = 1.35 - 1.46 (1 H, m)^a^, 1.47 - 1.58 (1 H, m)^b^, 1.93 - 2.04 (1 H, m), 2.13 - 2.22 (2 H, m)^a^, 2.22 - 2.30 (2 H, m)^b^, 2.56 (1 H, dt, *J* = 14.3, 7.0 Hz), 2.71 (1 H, ddd, J = 13.2, 7.2, 5.6 Hz), 2.90 - 2.94 (1 H, m), 3.23 (3 H, s), 3.27 (1 H, dd, *J* = 8.6, 6.3 Hz)^a^, 3.37 (1 H, dd, *J* = 8.5, 6.4 Hz)^b^, 3.71 (1 H, qd, *J* = 7.7, 2.6 Hz), 3.76 - 3.89 (2 H, m), 4.78 - 4.92 (1 H, m), 6.10 - 6.26 (1 H, m), 6.82 - 6.95 (4 H, m), 7.18 - 7.25 (3 H, m), 7.30 - 7.40 (3 H, m). **^13^C NMR** (CDCl_3_, 101 MHz): δ = 31.9^a^, 31.9^b^, 35.9, 37.6, 39.2^a^, 39.2^b^, 39.8^a^, 39.9^b^, 50.9, 67.6, 72.8^a^, 72.8^b^, 126.9, 127.2 (2 C), 128.2, 128.4 (2 C), 129.2 (2 C)^a^, 129.2 (2 C)^b^, 129.8 (2 C), 136.2, 142.3, 170.7, 171.5 (a, b = signals belonging to one diastereomer). **ESI-MS** [M+H]^+^ calculated for C_22_H_27_O_3_N_2_: 367.5; found: 367.4.
